# Migrant entrepreneurship support in Europe: a PRISMA systematic literature review

**DOI:** 10.12688/f1000research.139670.2

**Published:** 2024-01-22

**Authors:** Dimitris Polychronopoulos, Anh Nguyen-Duc

**Affiliations:** 1 University of South-Eastern Norway, Drammen, Norway; 2 University of South-Eastern Norway, Bø, Norway

**Keywords:** Migrants, immigrants, entrepreneurship, integration, mixed embeddedness, entrepreneurship support

## Abstract

**Background:**

This systematic literature review (SLR) analyzes migrant entrepreneurship support in Europe through three research questions (RQs) to understand 1) migrant entrepreneur characteristics in the European context, 2) challenges encountered by migrant entrepreneurs in European host countries, and 3) policies supporting migrant entrepreneurship in Europe. This review addresses gaps in current knowledge in academia as well as issues that policymakers and practitioners face when addressing migrant entrepreneurship support.

**Methods:**

This SLR employed a search protocol to retrieve published sources from 1970 to 2021, via Scopus (27 March 2022) and Web of Science (7 April 2022). Inclusion criteria targeted migrant entrepreneurship support studies while exclusion criteria eliminated domestic migration and non-European contexts. The authors worked iteratively, aligning the data with the RQs to reduce bias, and adapted Bourdieu's forms of capital to create an analytical framework for the sources included in the SLR, with a table for each RQ to synthesize relevant data for analysis.

**Results:**

The review examined 91 peer-reviewed papers, with a focus on migrant entrepreneurship support in Europe, covering characteristics, challenges, and support policies. It classified migrant entrepreneur challenges and characteristics into financial, human, and social capital, as well as external factors. Common challenges include the local culture and language, network, funding, and adapting to local business practices. Migrant entrepreneurs' stability relates to time in the host country and local language proficiency and reflects past entrepreneurial experience and education. Supportive mechanisms involve local networks, financing, and mentoring.

**Conclusions:**

The SLR's limitations encompass possible oversight of pertinent studies, along with potential bias in data extraction, analysis, and subjectivity due to thematic analysis. Nonetheless, the findings suggest the following research agenda for migrant entrepreneurship support: evaluating and enhancing human and social capital, sharing information, designing support programs, addressing in-group/out-group bias in support programs, and exploring bottom-up migrant entrepreneurship support approaches.

## 1. Introduction

### 1.1 Rationale

Migrant entrepreneurship in Europe has gained increasing attention in recent years as a means for individuals to pursue economic opportunities and contribute to the development of their host communities ( Aki Harima and Freudenberg, 2020; Kachkar and Djafri, 2021; Lyon, Sepulveda, and Syrett, 2007). As migrants comprise an increasing proportion of the population in Europe, they are seen to be more likely to be entrepreneurial than the native population ( Irastorza and Peña, 2007), especially in certain countries, such as Belgium, Denmark, France, Norway, Sweden, and the United Kingdom ( Kushnirovich, Heilbrunn, and Davidovich, 2018). Migrant entrepreneurship can be defined as the process of starting and running a business venture by an immigrant in a host country ( Communities, 2003), can have a positive impact on the economy of the host country ( Meister and Mauer, 2019) and be a source of innovation, economic growth, and social cohesion, as well as a means of integration and personal development for the entrepreneur ( Eraydin, Tasan-Kok, and Vranken, 2010). In addition, migrant entrepreneurship can build international connectivity and reinforce economic activity for the host country ( Kourtit, Nijkamp, and van Leeuwen, 2013); Backman, Lopez, and Rowe (2021) see under-utilization of immigrants’ skills as waste of resources. Beyond the above considerations of migrant entrepreneurship, Europe is a unique area to study and merits a special focus for four main reasons: the first is the right to free movement of European Union (EU) citizens ( Collett, 2013) and within the European Economic Area (EEA) ( Peers, 2000); the second is the application of the Dublin agreement in processing asylum applications ( Angenendt, Engler, and Schneider, 2013; Murray and Longo, 2018), combined with the dominance of EU/EEA amongst Western countries in receiving asylum applications, with more than 60% of the applications ( Hatton, 2020). A third consideration is Europe’s position in 2015 concerning its ability to accommodate a significant influx of new arrivals from outside the EU/EEA, along with the resulting effects ( Scipioni, 2017). Finally, scholars in Europe have recognized the need for a broader societal framework to understand migrant entrepreneurship, in order to account for the unique opportunity structures in Europe, by considering the role of economic and political factors on migrant entrepreneurship ( Kloosterman and Rath, 2018).

An increase in people moving to Europe coincides with the demographic trends that require immigration to fulfill labor needs and support the pensions of those who will soon be retiring and those who have already retired ( Marois, Bélanger, and Lutz, 2020). Some new arrivals express an interest in becoming entrepreneurs. For example 7% of Ukrainian refugees who settled in Norway report that they aim to become entrepreneurs ( Hernes, Deineko, Myhre, Liodden, and Staver, 2022) and the European Commission has incorporated refugee entrepreneurship into its *2020 Entrepreneurship Action Plan* and the European Union Qualification Directive 2011/95 (article 26 and 34) states that EU members must allow access to self-employment and consider the specific needs of refugees within integration programs (de Lange, Berntsen, Hanoeman, and Haidar, 2021). This SLR sees a knowledge gap surrounding the literature to support migrants of all kinds in their entrepreneurship journey, whether refugee or others. Considering that a literature review on entrepreneurship support by Ratinho, Amezuca, Honig, and Zeng (2020) shows a lack of defined evaluation of the outcomes of entrepreneurial support, this SLR aims to contribute by adding conceptual clarity to the body of literature on migrant entrepreneurship support in Europe. Therefore, with the objectives in the next section, we seek to map out the research issues on this topic. The need for this SLR is based on the context of the demographic trends of Europe, the increase in immigration to Europe, the desire of a portion of the immigrants to Europe to become entrepreneurs, and a lack of understanding surrounding the effectiveness of migrant entrepreneurship support in European contexts ( Chliova, Farny, and Salmivaara, 2018; De Noni and Ganzaroli (2013); Lillevik and Sønsterudbråten, 2018), as well as a lack of trust by some migrants in government-run migrant entrepreneurship support programs ( Ram, Theodorakopoulos, and Jones, 2008; Mwaura *et al.*, 2019), and the call by Dheer ( 2018) for scholars to study policies for their impacts on migrant entrepreneurship.

### 1.2 Objectives

Given that the literature on migrant entrepreneurship is fragmented, with studies focusing on various aspects of migrant entrepreneurship such as ethnic enclaves, mixed embeddedness, motivations, challenges, and outcomes ( Ilhan-Nas, Sahin, and Cilingir, 2011), a systematic review of the existing research will help synthesize the current knowledge on migrant entrepreneurship support in Europe and identify gaps for future research. Such a review will not only provide a comprehensive overview of the field, but also serve as a useful resource for policymakers, practitioners, and researchers interested in understanding and supporting migrant entrepreneurship in Europe.

This study aims at synthesizing current knowledge on migrant entrepreneurship support in Europe, by understanding the key issues that migrants in Europe face in their entrepreneurship endeavors and how these issues relate to key policy-making decisions. The study seeks, to develop a conceptual framework and identify research gaps that call for future inquiry. From the above objectives, we will address three research questions (RQs) in this study, which we divide into characteristics, challenges, and support mechanisms.

1. RQ1: What are the characteristics of migrant entrepreneurs investigated in primary studies, in the European context?
2. RQ2: What do we know about challenges that migrants face as entrepreneurs in European host countries?
3. RQ3: What do we know about reported policies as support mechanisms for migrant entrepreneurship in the European context?

To the best of our knowledge, this work offers a most up-to-date and comprehensive view to discern migrant entrepreneurship support in the European context and positions the issues uncovered as the foundation for policymaking. Existing systematic literature reviews (SLRs) have called for further work to (1) identify governmental and support policies for migrant entrepreneurs ( Malerba and Ferreira, 2020), and their impact on immigrant entrepreneurship ( Dheer, 2018), (2) synthesize the potential impacts of regional contextual factors ( Dheer, 2018) and country contexts ( Dabić *et al.,* 2020), and (3) focus on specific geographical areas. Our current study is the latest and most exhaustive secondary study that contributes to all these points.

The paper is structured as follows: Section 2 presents the background, including definitions of relevant terms. Section 3 presents our research approach. Section 4 contains our findings. Section 5 shares our discussions and recommendations, and Section 6 concludes the paper.

## 2. Background

### 2.1 Definitions of Migrant entrepreneurship

To provide further context surrounding the phenomena studied, it is important to define both the terms, ‘migrant’, and ‘entrepreneurship’. We start by clarifying that this study is not concerned with internal migration within the borders of a single country. The definition of ‘migrant’ in much of the academic literature, means that somebody is: 1) foreign-born and 2) a non-citizen ( Gimeno-Feliu *et al.*, 2019). Since the foreign-born can often acquire citizenship in their host country, foreign-born is a more suitable definition for this research. However, the non-citizen aspect is also important, since in addition to becoming citizen of a new country, an individual can be born abroad due to parents’ stay abroad; citizenship in country ‘A’ may also extend to the grandchildren of people who left country ‘A’ for country ‘B’, and in some cases, even to further generations back.

Some of the literature refers to migrant entrepreneurship ( Berntsen *et al.,* 2021; Hagos, Izak, and Scott, 2019; Sinkovics and Reuber, 2021; Solano, 2021; Szczygiel, Nunes, and Ramos, 2020), while some refers to immigrant entrepreneurship. ( Abbasian and Yazdanfar, 2015a; Bolzani and Boari, 2018; Glinka and Hensel, 2020; Murphy, Bogue, and O'Flaherty, 2020; Širec and Tominc, 2017; Yazdanfar and Abbasian, 2013). For this paper, both ‘migrant’ and ‘immigrant’ literature are suitable, if the condition of foreign-born is met. Throughout this article, the authors will refer to migrants and immigrants, and sometimes switch from one term to the other. This is because of the 91 sources that are included in this systematic literature review, the sources themselves may sometimes be based on the term migrant, while at other times based on the term immigrant. For this study's sake, both terms are relevant, and that is why we include both terms throughout the study. Dabić *et al.,* (2020) reported that ‘migrants’ are people who move to a different country from their usual residence, for a period of at least 12 months and that ‘immigrants’ are born abroad. Dheer (2018) emphasizes that the definition of immigrant denotes a move that is permanent and not transitory. However, for this study, we are interested in the phenomenon of migrant entrepreneurship support regardless of whether the intention is to stay permanently in the host country.

In a review of the literature on self-employment and entrepreneurship, Szaban and Skrzek-Lubasińska (2018) position self-employment within the entrepreneurship paradigm. Amongst authors included in our review, definitions of entrepreneur include creating or establishing a business ( Glinka and Hensel, 2020; Širec and Tominc, 2017; Solano, 2021), owning a business ( Abbasian and Yazdanfar, 2015; Yazdanfar, Abbasian, and Brouder, 2015), managing one’s one business ( Szczygiel *et al., 2020* *)*, and having an aim to succeed at business ( Hagos *et al.,* 2019). Given that migrants who are just getting started in their host countries and seeking support from government entrepreneurship initiatives may not have established a business yet, the broader definition by Hagos *et al.*, (2019) seems the most comprehensive for this study.

Since this research aims to study phenomena of migrant entrepreneurship in a broad sense of business activity and migration across national borders, we recognize that migrants may fall into categories that include the following: moving to the country of citizenship inherited from a parent or grandparent, fleeing a warzone as a refugee, joining a partner in a different country with whom they are in a romantic relationship, repositioning to the host country as an employee, trailing spouse, son or daughter, and staying on as an entrepreneur, seeking better economic conditions, seeking political asylum, adoption as a child by new parents in the host country, and staying in the host country after having studied abroad. Dheer (2018) defines immigrant entrepreneurs as those who “identify, create and exploit economic opportunities to start new ventures in their destination nations” (p. 558).

Some of the literature engages in a narrow focus of migrant entrepreneurship; examples include articles that exclusively study tech startups as well as a single case study of a cheese factory. However, there are many categories of entrepreneurial ventures, and they can overlap. This literature review is interested in all forms of business classifications to encompass entrepreneurship; examples include: business to business, business to government, business to consumer, software tech, hardware tech, deep tech, and consulting services, with offerings as either a service or a physical product. Another context is the locations in the studies we will be reviewing. The academic literature includes primarily studies with advanced economies ( Dheer, 2018), although Duan, Kotey, and Sandhu (2021) note a trend since 2012 for research to include emerging economies. Our study aims to gather knowledge from the overall European context, regardless of how advanced the economy is.

### 2.2 Common characteristics of migrant entrepreneurship

Existing literature suggests that immigrants to a new country are lacking in several areas, such as credentials, cultural understanding, and language skills, which create challenges that may push them to seek to earn a living by becoming entrepreneurs ( Ensign and Robinson, 2011). Li (2000) also notes that immigrants are often driven into entrepreneurship due to obstacles finding employment in their host countries. In addition, Cobas (1986) and Evans (1989) suggest that immigrant groups arriving in some countries have a greater tendency to become self-employed, while some groups of immigrants tend to make more successful entrepreneurs than others ( Zhou, 2010). Given that successful entrepreneurship activity drives economic growth ( Carree and Thurik, 2010; Schumpeter, 1949), that immigrant entrepreneurs can help the economies of their host countries ( Barth and Zalkat, 2021), that the rate of entrepreneurs is often higher for immigrants than amongst native populations ( Dheer and Lenartowicz, 2020), and that foreign-born entrepreneurs bring unique resources yet face specific challenges that the native population does not ( Bijedic and Piper, 2019), it follows that policymakers have an interest in helping improve the likelihood of entrepreneurial success for immigrants.

### 2.3 Existing literature reviews

To understand the need for a new systematic literature review on migrant entrepreneurship, we first conducted an *ad hoc* review of six systematic literature reviews on this topic. We examined the background information to help develop our research questions. The six literature reviews are by: Aliaga-Isla and Rialp (2013), Dabić *et al.,* (2020), Dheer (2018), Duan *et al.,* (2021), Malerba and Ferreira (2020), and Sithas and Surangi (2021). Table 1 displays the number of studies reviewed, the years covered, the main goals establishing prior to conducting the reviews, key findings, and the authors’ recommendations. Aliaga-Isla and Rialp (2013) extracted primary studies’ objectives, theoretical frameworks, and methodologies, finding that most studies took place in the United States, followed by Europe and Oceania, and that studies from Germany and the Netherlands were dominant for Europe and that Europe had a greater prevalence of empirical studies. Dabić *et al.,* (2020) plot the studies they reviewed into four quadrants by geographical scope, major research themes, methodological approaches, and theoretical approaches, with Europe leaning toward more qualitative studies and the use of embeddedness theory. Dheer (2018) outline the factors influencing the outcomes of immigrant entrepreneurship at the micro, meso, and macro levels, in addition to the process and its outcomes. In addition to the details covered in Table 1, Duan *et al.,* (2021) extracted elements including article type, citation rate, methodology, and extra context (such as gender or generation). The study documented a trend in 2012 for emerging economies to conduct research in immigrant entrepreneurship, as well as a trend starting in 2007 for publications in business and entrepreneurship to cover the phenomenon. They find that push-pull theory dominates the field, with its application in 28 of the 62 articles. Malerba and Ferreira (2020) make a novel contribution with a larger search scope (adding Scopus) and the new focus on the identification and exploitation of opportunities by immigrant entrepreneurs, which Dheer (2018) identified as a key issue for future research. Sithas and Surangi (2021) find that most studies concentrate on immigrant communities in the host countries rather than on ethnic differences inside the native country. They reported a dominance of literature of immigrants from African and Asian countries to Western and European countries and that the dominant theories in the literature are community of practice theory, cultural theory, effectuation theory, and ethnic enclave theory, and demonstrate the interactions of these theories between individual members and the greater community, noting that the ethnic enclave extends support to the individual, while also accounting for factors that enhance the individual’s personality traits. There appears to be some overlap with ethnic enclave theory and Community of Practice Theory and Cultural Theory, since all three postulate that the ethnic group has an impact on the individual member’s personality traits, with effectuation having its place to explain the back-and- forth between the individual and the community ( Sithas and Surangi, 2021).

**Table 1.** An overview of six literature reviews on migrant entrepreneurship.

Authors	Number of Studies reviewed	Years covered	Goals	Findings	Recommendations
Aliaga-Isla and Rialp (2013)	45	1985-2011	To gain an overview of objectives, theoretical frameworks, and methodologies	Mostly individual level of analysis with deductive studies, with human capital, social cognitive, disadvantage and national/cultural theories dominating the literature	Need more theory-building and qualitative studies
Dabić et al. (2020)	514	1991-2018	To gain an overview of theories, methods, and contexts	Creation of a conceptual framework for home and host countries, with antecedents, decisions, and outcomes and where theories apply in each case	Need for interdisciplinary approaches and to adopt different theoretical frameworks, to use multi-level methods and explore new country contexts
Ratan J. S. Dheer (2018)	69	1980-2016	To delineate the boundaries /outline contributions /identify gaps/catalogue journal disciplines	Disciplines of the most frequent journals covering the topic are: entrepreneurship, ethnic studies, and social science; steady increase in articles cited over time	Need to investigate the effects of human capital, ethnic ties, group attributes, and regional contextual factors, and to study regulatory environment and policies for their impacts on immigrant entrepreneurship
Duan et al. (2021)	62	1993-2020	To understand the determinants; home-host country, methods, time, study type, typology, journal discipline	Creation of an analytical framework with the overview of determinants of immigrant entrepreneurship motivation and classification as either individual or environmental push-pull factors, depending on immigrant entrepreneurial motivation	Need to explore transnational immigrant entrepreneurship and to study determinants in the immigrant entrepreneurial ecosystem and how motivation impacts strategies and outcomes
Malerba and Ferreira (2020)	67	1998-2019	To understand the strategies that immigrant entrepreneurs apply to create, maintain, and grow their businesses	Highlights ethnic enclaves to create competitive advantages prior to mainstream market entry and expansion to other countries	Need to understand opportunity recognition, business creation, adaptation, survival strategies and influences of ethnicity on immigrant entrepreneurial strategies
Sithas and Surangi (2021)	174	2010-2020	For citation analysis to classify key areas of research and thematic analysis to discover themes	Identified seven themes: 1) immigrant studies, 2) ethnic entrepreneurial motivations, 3) ethnic startup process, 4) motives of ethnic business success, 5) failure factors, 6) unique challenges, and 7) favorite strong ties	Need to explore ethnic differences within the native country and to research in Asian countries, plus sociological perspectives of established firms, racism, religion, network, as well as qualitative studies and theory exploration

The six literature reviews we examined provided insight into the topics, theoretical perspectives, empirical contexts, and methodologies employed in current research on migrant entrepreneurship, as well as identified gaps for future research. While there is a growing body of literature on migrant entrepreneurship in Europe, these reviews have not limited themselves to the topic of support, nor have they extensively covered the European context, which are the distinctions of our review. In addition, both Dheer (2018) and Malerba and Ferreira (2020) emphasize the importance of the impact of policies on migrant entrepreneurship, yet none of the literature reviews set out to do so. We see that immigration is getting more attention from both policymakers and scholars in Europe ( Dheer, 2018). While new ventures that immigrants establish in their host countries can help lead to economic development and a decrease in unemployment, we also realize that some of the immigrants get stuck in low-margin businesses, while others fail at their businesses ( Dheer, 2018). Considering the above, our literature review will fill the gaps in existing research by examining the characteristics and challenges of migrant entrepreneurs in Europe, as well as the policies and support mechanisms that impact their experiences in host countries and how ecosystems influence these policies.

## 3. Methods

**Figure 1.**
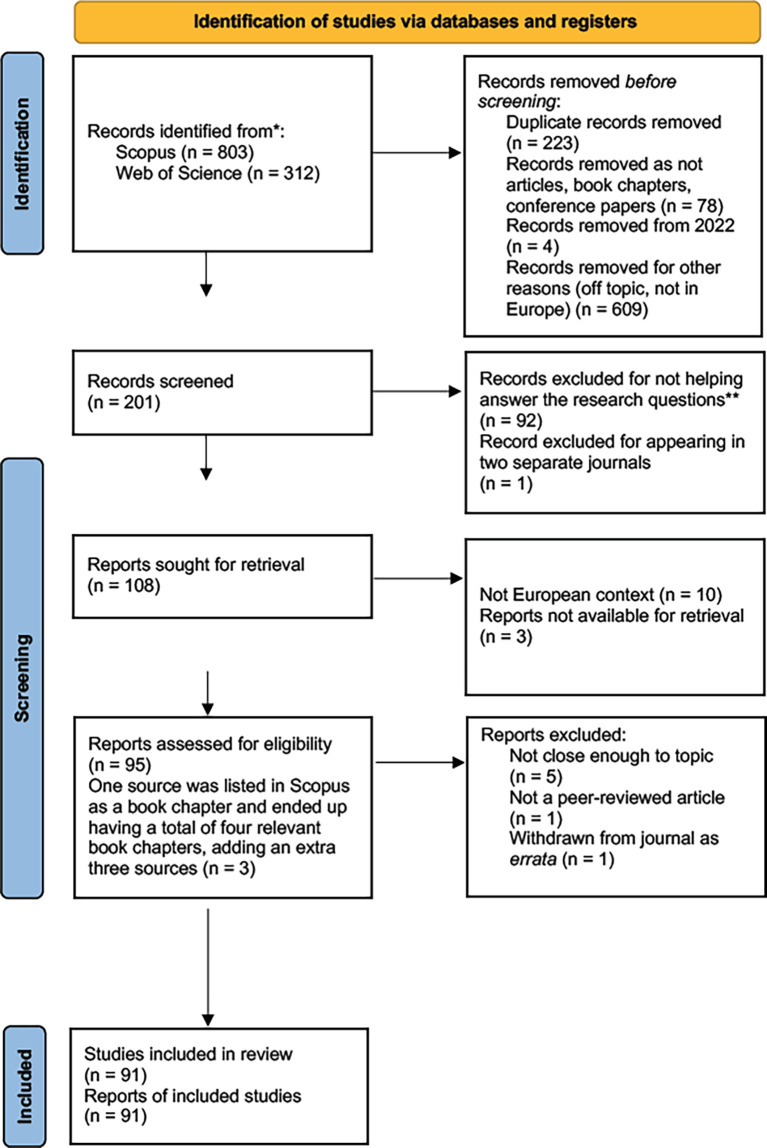
The study selection process.

We adopted an existing well-known methodological framework to ensure that the systematic literature review would provide a comprehensive and structured synthesis of available research on migrant entrepreneurship support in the European context, by following the steps described by Kitchenham (2015). First, we developed the search protocol. Second, we identified the inclusion and exclusion criteria for relevant publications. Third, we performed an in-depth search for studies, followed by critical appraisal, data extraction and synthesis of past findings. The next sub-sections describe in detail the previously mentioned stages. We also adopted the PRISMA (Preferred Reporting Items for Systematic reviews and Meta-Analyses) guideline for reporting systematic reviews ( Page *et al.,* 2021). We use the PRISMA template ( Page *et al.,* 2021) to report the process and outcomes of each step in the search process Along with these guidelines, Figure 1 displays the PRISMA 2020 flow diagram for the study selection process, from identification of studies, to screening of studies, and inclusion of studies, while the PRISMA checklist and PRISMA abstract checklist are publicly available with this report ( Polychronopoulos and Nguyen Duc, 2023). See Table 11 for the links to the documents included in this study.

### 3.1 Search protocol

The eligibility criteria helps with the search strategy to derive relevant literature from the main RQs, with our search string has three parts: MIGRANT (C1), ENTREPRENEUR (C2), and SUPPORT (C3). Under this search string, the idea is to cover migrant under diaspora, refugee, immigrant, exile, displaced or asylum, and to cover entrepreneurship under various forms and include self-employment, startup, venture, new business, enterprise, new firm, or similar, and to cover support under training, as well as forms of acceleration or incubation. The synonyms to these terms were identified in the context of either information systems or entrepreneurship research by interviewing field experts. We presented the list of synonyms related to migrant entrepreneurship support to our colleagues. Two of these experts came from the information systems domain, while one had a background in entrepreneurship. We asked them to contribute to the list of synonyms based on their knowledge. Each conversation occurred individually, informally and lasted for circa ten minutes. Their feedback was documented and resulted in a revised list of synonyms. The two authors conducted several trial searches to adjust the scope of the search string, so that we do not include many irrelevant studies from different research fields. The most important information is the supporting mechanisms that were provided to address challenges that migrant entrepreneurs confront. On the other hand, we want to include as many studies as possible. After several trials, we culminated with the list of search words as shown in Table 2. The search string is formed as the formula: C1 AND C2 AND C3.

**Table 2.** Synonyms to key search words.

Search part	Main term	Explanation	Synonyms
C1	migrant	people living for a year or more outside the country of their citizenship, their country of birth, or the country where they grew up.	“Migrant” OR “diaspora” OR “refugee” OR “immigrant” OR “exile” OR “displaced” OR “asylum”
C2	entrepreneur	People who are either self-employed or who established a business as their primary way to earn a living	“Entrepreneur*” OR “self-employ*” OR “startup” OR “venture*” OR “start-up” OR “new business*” OR “enterprise” OR “new firm*” OR “new compan*” OR “NTBF” OR “new technology-based firm” OR “new technology based firm”
C3	support	An initiative to teach/support people a set of abilities and skills	“Training” OR “Support” OR “incubat*” OR “accelerat*”

Our linguistic capabilities meant that we could include studies published in the following languages: Danish, English, French, Greek, Italian, Norwegian, Portuguese, Spanish, and Swedish. However, nearly all articles in the search were published in English, with a few appearing in Spanish. By the time we had concluded the inclusion/exclusion process, we had one article in Spanish and 90 in English. The authors considered several electronic databases, including Scopus, ISI Web of Science, IEEE Explore, Current Contents, Kluwer Online, Computer Database, Science Direct, Springer Link, Inspec and ACM Digital Library. Considering the popular databases within the entrepreneurship research, previous experiences of reviewers, flexible formulation of search strings with unlimited clauses and easily exporting paper lists in various formats, we decided to select Scopus and ISI Web of Science. The search ranges from 1970 to March 2022. We screened the sources based on the title, abstract, and keyword metadata to help us select studies relevant to our RQs.

### 3.2 Inclusion and exclusion criteria

To prepare for the next stage in the process of the eligibility criteria, we developed the following inclusion and exclusion criteria:

Inclusion criteria:

• IC1 - the paper should investigate migrant entrepreneurs as the main research topic
• IC2 - the paper should explore either challenges migrant entrepreneurs face in their host country, policy on the topic, or both
• IC3 - the paper should discuss how to train entrepreneurs, factors for migrant entrepreneurship success, or both

Exclusion criteria:

• EX1 - the paper does not investigate migrant phenomenon transnationally
• EX2 - the paper does not investigate migrant entrepreneurship as a primary topic
• EX3 - the paper does not investigate entrepreneurship topics

For completeness, we also excluded papers that were published in 2022 because it was March of that year at the start of the search, and we could not fully represent 2022 within the review.

### 3.3 The study selection process

Concerning information sources, the only databases used to search for sources were Scopus and Web of Science. The beginning of our search yielded 803 results in Scopus on 27 March 2022 and 312 results in Web of Science on 7 April 2022. After merging the results to eliminate the 223 duplicate papers that appear in both Scopus and Web of Science, we were left with 803 Scopus sources and 89 results from Web of Science, for a total of 892 results. We conducted the selection process as shown in Figure 1.

Remove non peer-reviewed documents: after removing the duplicates, we removed four titles published in 2022. We then removed 78 records for not being in the category of articles, book chapters, or conference papers. This left us with a total of 810 records for further screening.

Prior to applying the inclusion and exclusion criteria, the two reviewers performed pilot runs, i.e., pretest, to improve homogeneity. The aim was to ensure that the reviewers had the same interpretation of the inclusion and exclusion criteria, which meant that there was a good understanding of the type of studies that needed to be included and excluded. The first pilot consisted of 20 papers, which the authors chose for their mix of theory, entrepreneurial incubation, policymaking, migrant integration, and refugee topics in their titles. At this stage the authors were not yet focused on excluding non-European settings. The reviewers were able to retrieve 19 articles of the 20 articles and held meetings to discuss the them after reading through them. Fleiss’s kappa for agreement on inclusion in the review was 0.84 for the two reviewers ( Fleiss, 1971). The kappa value between 0.81 and 1.00 represents an almost perfect agreement ( Fleiss, 1971), suggesting a very good agreement among the authors. In case a decision could not be made by two reviewers, we kept on-going discussion until consensus was reached. Eleven of the studies made it past the original pilot review and eight of them did not. Primary studies use different research designs, data collection and analysis methods, which leads to a threat of validity of synthesis process. To reduce the variety in primary studies, we used an extraction form, which is driven by the research questions. The form had been initiated by studies in the initial pool and validated during the trial search process. Only studies that addressed at least two RQs made it through the first pilot review.

The next step was to eliminate sources that did not cover the European context. At this step, five sources made it through to be amongst the total of 91 studies included in this SLR. They are the following sources: 1) Solano, 2021, 2) Berntsen, de Lange, and Kalaš, 2021, 3) Harima, Freudenberg, Halberstadt, and Hanoeman 2020, 4) Bruzelius, 2020, and 5) Evansluong and Ramírez-Pasillas, 2019.

Select by title and abstract: we removed articles that clearly have no connection to the topic of migrant entrepreneurship, as well as those articles which clearly were beyond the scope of the European context. Here we excluded articles from the Kazakh, Russian and Turkish local contexts as well, given that most of the landmass of these three countries falls in Asia rather than in Europe, and given our expectations that the conditions surrounding migrant entrepreneurship support in these three countries would differ significantly from those of the rest of Europe, considering not only their geography, but their cultural traditions and political structures. By considering the three research questions as we moved forward, we could identify documents that did not cover aspects of migrant entrepreneurship such as characteristics, policies, support, challenges, and success factors. At this stage the authors reviewed the titles and abstracts to decide upon which articles to include and which to exclude. Figure 1 below displays the study selection process.

Geographical context was not always evident from the titles and journals. Thus, the authors recognize that in the next step they would need to check for geographical context in the abstracts. This process allowed us to eliminate 609 sources, leaving us with 201 documents for further scrutiny. At this stage the authors reviewed the titles and abstracts to decide on which articles to include and which to exclude.

Select by full text: when the selection could not be determined with abstract and titles, it was accomplished by reading full texts. Both authors assessed the papers and sought to understand migrant entrepreneurship support as an emerging line of research in the business literature and to understand the success factors and moderators of success. We looked for articles that would help answer the research questions based on the following list:

1. How to stimulate entrepreneurial activity
2. How to improve likelihood of success (decrease early-stage failure)
3. To understand and identify barriers to growth
4. How to incorporate the needs of entrepreneurs in developing and support systems
5. Policymaking for migrant entrepreneurs
6. Migrants becoming self-sufficient either as entrepreneurs or as employees
7. Creating the entrepreneurial aptitude for migrants
8. Understanding entrepreneurial support organisations

This stage of the review brought us to 108 articles for further scrutiny, with all publication years known and the oldest being from 1994, while 62 of the articles were published from 2018 to 2021. Sweden was the most frequently appearing European country, with 17 articles, followed by Italy at 11, and the United Kingdom at 10. Some articles also covered multiple countries and included European and non-European contexts. We also discovered a duplicate article within Scopus, with the same title, but published in two different journals. ‘A new career in a new town: Entrepreneurship among Syrian refugees in Germany and the Netherlands’ *,* in both the International Journal of Entrepreneurship ( Johnson and Shaw, 2019a) and the Journal of Legal, Ethical, and Regulatory issues ( Johnson and Shaw, 2019b).

Further evaluation and selection: the next step was to read through all the articles whose geographical contexts were still unknown, and to include them only if they covered the European context. After sorting through the articles where the geographical context was not available in the abstract, we were able to eliminate another 10 sources. This brought us to 98 articles for further scrutiny with an updated list providing geographical context, with Sweden, UK, Germany, and Italy appearing most frequently. At this stage we went through further examination on the inclusion criteria and found that five of the articles were not close enough to the topic, which are:

1) ‘Migrant women entrepreneurs and emotional encounters in policy fields’ ( Webster, 2020) had a narrow focus on emotion and didn’t seem to define ‘policy fields’
2) ‘Social innovation in Refugee Support: Investigating Prerequisites Towards a Conceptual Framework’, ( McNally, Apostolopoulos, and Al-Dajani, 2020) consisted of five chapters with focus on social innovation, but no clear focus on characteristics, challenges, and policies.
3) ‘Entrepreneurial cultural affinity spaces: Design of inclusive local learning ecosystems for social change, innovation, and entrepreneurship’ ( Savva, Souleles, and Ferreira, 2020), was classified as a conference paper, but turned out to be only lecture notes
4) ‘Factors driving the share and growth of Chinese entrepreneurship in Italy’ ( Apa, Noni, and Ganzaroli, 2020}, had a very narrow focus on Chinese entrepreneurs in the fashion industry in Italy, with little focus on characteristics and policy
5) ‘Ubicación espacial de los negocios étnicos en Almería. ¿Formación de enclaves económicos étnicos?’ ( Garrido and Olmos, 2007), does not focus on challenges, characteristics, or policy.

We also detected one study which was not a peer-reviewed article and one study that was withdrawn from the published journal. Furthermore, for three articles we cannot access their full text from our universities, which are 1) ‘Diaspora Africans and entrepreneurial characteristics: A focus on Nigerians in the UK’, 2) ‘Self-employment work and small enterprises as channels of integration for immigrants: The case of the Province of Trent’, and 3) ‘Recent refugee migrations to Western Europe: asylum seekers and refugees in Italy and Greece’. In addition, our Scopus search included a book chapter from *Female Immigrant Entrepreneurs: The Economic and Social Impact of a Global Phenomenon* ( Halkias *et al.,* 2016b). After borrowing the book from the university library system, it emerged that the book contained four chapters which are relevant to this SLR, given that each separate chapter is devoted to a specific country context: Cyprus, Greece, France, and the United Kingdom. By adjusting this from single source to four separate sources, it raised the number of sources by three.

At the end of this step, we emerged with the final set of 91 peer-reviewed sources, of which 90 are in English and one is in Spanish.

### 3.4 Data collection process, data items, and bias assessment

The authors created a spreadsheet to record specific data items from the articles. These data items include the database from which the article was located (Scopus or Web of Science), the year published, the title, the unit(s) of analysis, the authors, the retrieval method (PDF, print, or loan from library), the host country/countries, the reason(s) for inclusion criteria, the source title (name of book, conference, or journal), the characteristics of the migrant entrepreneurs studied, the home country/countries (if mentioned) and whether the study focused on refugees, the policies and support mechanisms to help migrants succeed as entrepreneurs, the challenges migrants face in their host countries, theoretical framework(s) and/or key concepts, research type (qualitative, quantitative, mixed methods, or theory exploration), the abstract, author keywords, and document type (article, book chapter, or conference proceedings). The authors collected the above details and manually input them into the spreadsheet. No automation tools were used in this process. The authors took an inductive approach to explore the input from the 91 articles and it emerged from content of the article collection that a breakdown of financial, human, and social capital would provide a framework for analysis in terms of answering the research questions, building a conceptual framework, and sharing key takeaways with the readers. The authors created a table for each form of capital: financial, human, and social, and filled it in with the details from the articles. The authors went through the tables together to discuss the contents of the tables. Through a process of several meetings, the two authors worked on the tables so they would be compact and clear enough for specific details to emerge that help answer the three research questions:

1. RQ1: What are the characteristics of migrant entrepreneurs investigated in primary studies, in the European context?
2. RQ2: What do we know about challenges that migrants face as entrepreneurs in European host countries?
3. RQ3: What do we know about reported policies as support mechanisms for migrant entrepreneurship in the European context?

The authors followed guidelines with a systematic approach to reduce individual bias and influence on the data selection process, so that others who follow the same search protocol and procedures outlined in this SLR’s sections 3.1, 3.2, and 3.3, should achieve the same results.

### 3.5 Synthesis methods and reporting bias assessment

The authors coded each source from S1 to S91 and created a table for each of the three research questions. Table 7 refers to RQ1 and includes human and social capital as the characteristics mentioned in the primary studies. Table 8 refers to RQ2 and includes financial, human, and social capital, as well as external factors as challenges for migrant entrepreneurs. Table 9 refers to RQ3 and includes financial, human, and social capital, as well as external factors as supporting initiatives and policies for migrant entrepreneurs in Europe. Here the ethnic group itself is sometimes mentioned as having its own social capital and this is noted in Table 9. Therefore, during the scanning of the full texts, the authors identified relevant paragraphs and texts and labeled them with codes that refer to the three research questions, with one table for each research question to record the relevant information from all of the 91 sources included in the SLR. In addition, the authors created tables to record the distribution by country, categories of journals, list of conferences and journals appearing at least twice.

As for reporting bias assessment, Elshater and Abusaada (2022) emphasize that bias can hinder researchers from creating a proper roadmap for their literature reviews, and define bias as favoring or disapproving a particular topic or author’s perspective. To address these issues, during the synthesis process, the authors worked closely together iteratively by regularly revisiting the research questions, the Tables 7, 8, and 9 where the data was recorded in answer to the research questions, and discussed and returned to the primary studies. The authors updated the tables several times and agreed to add external factors to Tables 8 and 9 (challenges and support) in order to complement the three forms of capital that emerged from the sources: financial, human, and social capital. In addition, the authors merged some of the references to Bourdieu’s forms of capital, such as cultural and economic capital into this framework, with economic capital appearing as financial capital in the tables and the analysis, while cultural capital appears either as human or social capital, depending on the context.

## 4. Results

### 4.1 Study characteristics

For each of the 91 studies included in this SLR, the authors created a unique identifier starting with the letter S for ‘study’ and a number from 1 to 91. The table displays the studies in chronological order from most recent to oldest, based on the order of appearance in the original Scopus search. The studies from Scopus are labeled with codes S1 to S86, followed by the same logic for sources from Web of Science, which received codes S87 to S91. Table 11 displays the codes S1 to S91, the authors and publication years, the host countries, the research type (qualitative, secondary qualitative, quantitative, mixed methods, or theory exploration), the type of document (article, book, book chapter, or conference paper), the home countries (if mentioned) and other key characteristics of the migrant entrepreneurs studied (whether the study focused on refugees or a specific gender), and theoretical frameworks used if mentioned explicitly.

**Figure 2.**
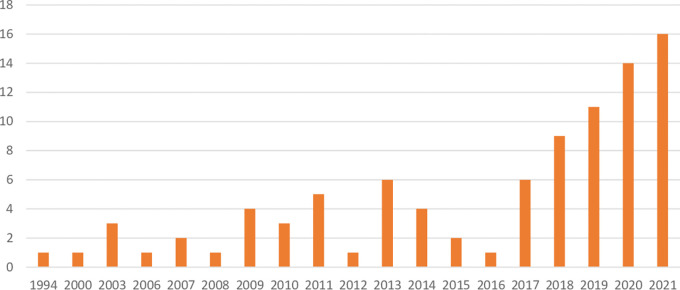
Distribution of primary studies over publication years.

As for the distribution of the selected primary studies across geographical context, Table 3 demonstrates that some countries have a greater research interest in the topic of migrant entrepreneurship support than others. Sweden is not only the country that appears the most frequently in the European literature on migrant entrepreneurship support, but it was also the first country to appear in chronological order within our 91 sources, with a publication dating from 1994 (See Figure 2 for the distribution of publication years). It is also of merit to note that Sweden has a significantly smaller population than the next five countries that top the list in sources about migrant entrepreneurship. This means that Sweden alone represents more than 17 percent of the weight in the results of the SLR, despite representing just more than 2 percent of the population of the European Union. It is also worth noting that 11 of the 91 studies consider more than one country. For example, Solano (2021)’s study is a review of measures that foster migrant entrepreneurship in countries across the European Union and Organization of Economic Cooperation and Development (OECD).

**Table 3.** Host countries reported in primary studies.

Geographical Context	Count
Sweden	16
United Kingdom	12
Germany	11
Multiple countries (two or more countries)	11
Netherlands	9
Italy	9
Spain	5
Greece	3
Portugal	2
Denmark	2
France	2
Finland	2
Norway	2
Ireland	2
Switzerland	1
Slovenia	1
Cyprus	1
**Grand Total**	**91**

Ten additional sources also cover multiple countries. 1) Wade (2020) studies the Fresh Start program and migrant education in Belgium, Netherlands and UK, 2) Glinka and Hensel (2020) provide an analytical framework from contexts of Belgium, Netherlands, Poland and USA, 3) Johnson and Shaw (2019) look specifically at Syrian refugees in Germany and the Netherlands, 4) Williams and Krasniqi (2018) explore human and social capital for migrant entrepreneurs from conflict zones and in several countries, mostly in the EU, 5) Eroğlu (2018) studies Turkish entrepreneurs in Western Europe, 6) Thandi and Dini (2010) use an interdependent perspective to examine secondary data of migrant entrepreneurship in Europe, 7) Van Delft, Gorter, and Nijkamp (2000) study comparative policy in various cities across six different European countries, 8) Širec and Tominc (2017) study growth determinants in 13 European countries, with an aim to cover less developed and more developed areas for comparative purposes, and 9) Bruzelius (2020) investigates local policy responses in Gothenburg, Hamburg, and Stockholm, for EU citizens moving to these locations. 10) Qin (2021) synthesizes the literature on cognitive dissonance, multiple embeddedness and hospitality to explain the implications on entrepreneurship and refugee business support in developed European economies.

In addition to discussing the geographical contexts of the host countries for studying phenomena surrounding migrant entrepreneurship support, we also wish to share some details about the nature of the studies in terms of gender, refugee status, and home country. Of the 91 studies, 41 were not specific to gender, refugees, or home country. In terms of home country focus, 23 of the studies were specific to a single home country, two of the sources studied two home countries, and 66 of the studies covered three or more home countries; in total 29 of the studies had a specific home country or set of home countries. The most frequent home country studied was Turkey, which appeared in seven of the studies, followed by Syria which appeared in four studies and Morocco and Pakistan which appeared in three studies each. Fourteen of the studies were specific to female entrepreneurs while one was a case study of a specific male Syrian refugee entrepreneur. An additional 16 sources investigated refugee entrepreneurs.

In Figure 2, we can see that the first publication is an outlier. This is the study by Najib (1994) on migrant entrepreneurship in Uppsala, Sweden, which was labeled in Scopus as an article but when borrowed from the university library system, it turned out to be a peer-reviewed booklet. We note that in the United States, there were studies published during the gap between 1994 and 2000, but none for Europe that met our inclusion criteria. This would demonstrate that while Sweden is at the vanguard of migrant entrepreneurship support in Europe, that Europe in general followed behind the uptick in migrant entrepreneurship support literature in the United States. Figure 2 shows several gaps in publications about migrant entrepreneurship support in Europe until 2006, at which point annual publications on the topic are the norm.

With Sweden as an outlier both in terms of its early arrival into the migrant entrepreneurship support literature, as well as with its volume of studies over time, it is worth looking into possible reasons for its prolific position. First of all, Sweden has high immigration rates compared to other European countries ( Evansluong and Ramírez Pasillas, 2019). Backman, Lopez, and Rowe (2021) state that Sweden had the largest share of asylum seekers between 2002 and 2013, which is one aspect that could help explain the large volume of literature covering the Swedish context. Secondly, Sweden has specific integration policies that emphasize new arrival’s first job ( Webster and Zhang, 2020), along with a rigid labor market and generous support system ( Backman, Lopez, and Rowe, 2021), with government organizations providing dedicated advice about entrepreneurship to immigrants ( Andersson, 2021; Högberg, Schölin, Ram, and Jones, 2016; Yazdanfar, Abbasian, and Brouder, 2015). A third factor is the change in immigration rules in 2015, where Sweden stopped recognizing credentials from the Middle East, thus resulting in higher rates of self-employment amongst impacted new arrivals ( Barth and Zalkat, 2021).

When considering Table 3 for geographical patterns, taking the top six countries, which each have at least five sources dedicated to a single country: Sweden, United Kingdom, Germany, the Netherlands, Italy, and Spain, it can be useful to compare them to gain further insights. For example, exclusive coverage of refugee entrepreneurship is absent from the examples from Spain, whereas an exclusive coverage of female entrepreneurship is absent from the examples from Germany. In-depth discussion on the topic of ethnic enclaves is also absent from the German sources, although ethnic communities and ethnic markets do provide context in the article by Bijedić and Piper (2019) and co-ethnic networks in the article by Meister and Mauer (2019) as well as ethnic communities, networks, and resources by Kontos (2003). Only Italy and the United Kingdom cover tech startups. The United Kingdom seems to have the highest rate of migrant entrepreneurship, with their rate being three times higher than native-born British ( Osaghae and Cooney, 2020). Investigations into entrepreneurship in the agricultural sector appeared in the studies from Germany, Italy, and Sweden, while research on internationalization or transnational entrepreneurship appeared only in the articles from Italy and the Netherlands. Both Germany and Sweden have government organizations exclusively focusing on migrant entrepreneurship, and both countries have literature expressing concern of the ‘othering’ of migrants, creating an in-group/out-group and expectations of inferiority of migrant entrepreneurship compared to non-migrant entrepreneurship ( Högberg, Schölin, and Ram, 2016; Mason, 2008; Rashid and Cepeda-García, 2021).

Of the 91 sources in this SLR, 81 are published in academic journals. Table 4 displays the categories of the journals where these articles have been published. We recognize that it is possible to classify some of the journals into more than one area and noticed that the greatest interest in the topic is from journals that focus on entrepreneurship. The authors developed their own journal categorization rather than using Scopus and Web of Science journal categories, because our coding recognizes the specific areas within management and social science fields to allow us to gauge publishing communities’ interest in migrant entrepreneurship support phenomena with more detail and to avoid an attempt at merging Scopus and WoS classification systems. For the journal classifications, which appear in Table 4, the authors checked specifically with the journal websites to read a description of the aims of each journal and created the categories based on these descriptions. While it is possible to classify some of the journals into more than one area, we noticed that the greatest interest in the topic is from journals that focus on entrepreneurship. Some disadvantages of our classification system are that we did not set clear boundaries since the categories overlap and are not mutually exclusive and there is some subjectivity in the authors’ interpretations of journal characteristics. However, we believe the advantages outweigh the disadvantages because our classification allows for more detailed insight into the publishing communities that have an interest in migrant entrepreneurship support phenomena, allowing for a deeper understanding of the focus and alignment of journals covering the topic. In addition, our classification method allows for greater specificity to allow for a more nuanced understanding of which academic fields are covering migrant entrepreneurship support.

**Table 4.** Category of journals appearing in the SLR.

Category of Journal	Count
Entrepreneurship/Business	35
Migration/Ethnic Studies	13
Government/Policy	12
Management (general)	5
Human Resources/Organizational Behaviour	5
Geography	4
Sustainability	3
Sociology	2
Gender Issues	1
Economics	1
**Total**	**81**

We see that most of the publications fit within the scope of the broader topic of management, which encompasses more specific areas such as entrepreneurship/business and human resources/organizational behavior. This means that 45 of the 91 sources are published in journals which fall under the topic of management, with 35 of the articles published in journals specific to entrepreneurship/business, five under human resources/organizational behavior, and five in journals that cover management issues on a broader level. The next two most common journal topics were immigration/ethnic studies and government/policy and immigration/ethnic studies with 13 articles and 12 articles respectively. The categories in Table 4 are not mutually exclusive, as there is much overlap between the categories. However, we aimed to be as specific as possible when larger volumes of articles are concerned, to demonstrate the range of scope from narrow to broad, based on the description of each journal. Table 4 covers only journal articles, which comprise 81 of the 91 sources in the SLR. Nine of the remaining sources consist of articles from conference proceedings and book chapters ( Bashir, 2018; Hartmann and Schilling, 2018; Mason, 2008; Ong and Freeman, 2017; Szczygiel *et al.,* 2020), with four of those book chapters published in Halkias *et al.* (2016b), and the final source is a peer-reviewed study from Uppsala University ( Najib, 1994).

In Table 5, we see a list of conferences and journals that have published at least two of the 91 sources included in this study. Note that the most frequently appearing source, with five articles, is the International Migration, which is published on behalf of the International Organization for Migration. This is a social science journal with a worldwide geographical scope that covers the gamut of policy regarding international migration concerns ( “International Migration Journal Overview,” 2023).

**Table 5.** List of conferences and journals with at least two papers included in the SLR.

Name of Journal or conference	Count of Source title
International Migration	5
Entrepreneurship and Regional Development	3
International Journal of Entrepreneurial Behaviour and Research	3
International Journal of Business and Globalisation	3
Journal of Enterprising Communities	3
Sustainability	3
Economic Geography	2
Proceedings of the European Conference on Innovation and Entrepreneurship, ECIE	2
Journal of International Entrepreneurship	2
Environment and Planning C: Government and Policy	2
International Journal of Entrepreneurship and Small Business	2
Ethnicities	2
Work, Employment and Society	2
European Countryside	2
Journal of Small Business and Enterprise Development	2
Contemporary Issues in Entrepreneurship Research	2
Small Business Economics	2
Innovation: The European Journal of Social Science Research	2
Journal of Ethnic and Migration Studies	2

### 4.2 Data extraction and analysis

To synthesize findings and categorize studies based on their scope, an analysis of the different research streams was performed. The first step was to identify the relevant information from each study, using the authors’ original terms. The key information was then organized in a spreadsheet to enable comparison across studies and translation of findings into higher-order interpretations. The following information were extracted: information of the authors and meta-data of the paper, research objective, research method, research type (qualitative/quantitative/mixed), theories and frameworks and how they were applied, the sample size, the instruments used (e.g., surveys, interviews, observations) main findings, characteristics of migrant entrepreneurs, their challenges, supporting policies (and their effect). Regular working sessions were conducted by the two co-authors during the data extraction and synthesis step.

**Figure 3.**
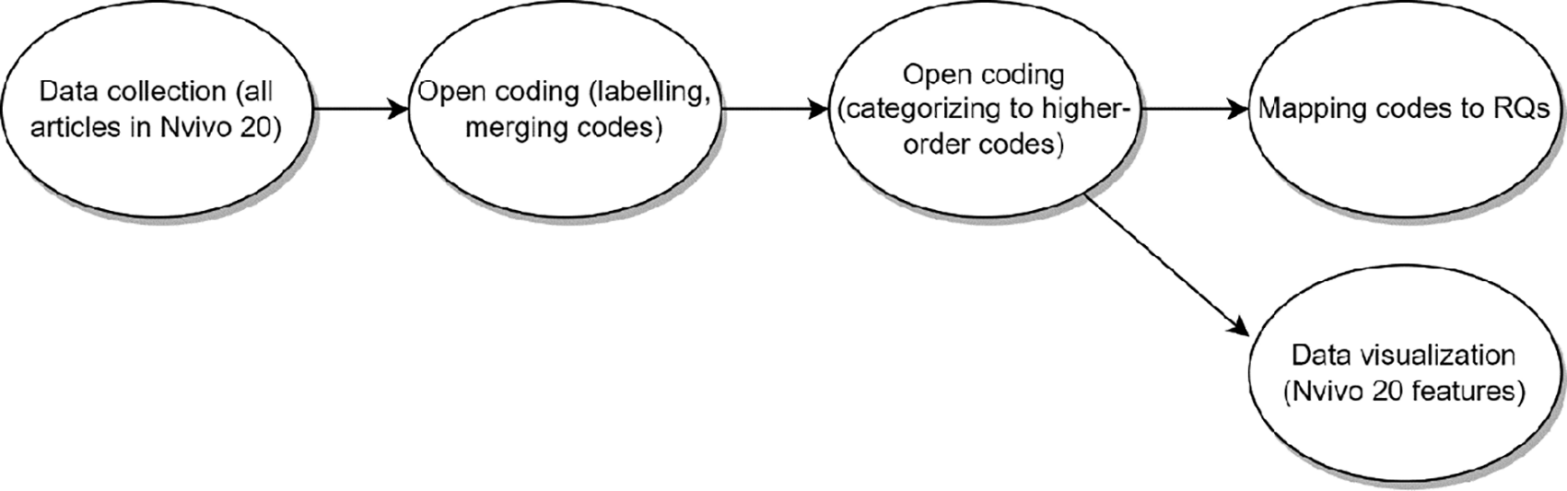
Data synthesis steps.

For extracting answers for RQs, we adopted a tailored thematic analysis with open coding, where the researchers decide which data to extract from the studies by following the research questions with the process to assemble the findings from the set of studies to draw conclusions ( Popay *et al.,* 2006). Of the goals for narrative synthesis outlined by Popay *et al.,* (2006) that we have identified as key for this SLR, are: 1) developing a preliminary synthesis from the 91 studies’ findings and 2) exploring relationships within the data. The authors went through all the papers, extracted the relevant text, and labelled them (open coding). The labels were then renamed and merged across articles, resulting in a united set of first-order code. After that, the codes were grouped in a higher-order code scheme, which are then mapped into answers for RQs. This process is outlined in Figure 3.

### 4.3 Threats to validity

One common threat to systematic literature reviews is not to discover all relevant studies. The reason not to cover all seed studies may be that the search ranges in multiple disciplines, such as Information Systems, entrepreneurship, and business research and innovation management. To reduce this threat, we adopted a quite generic search protocol to retrieve as many documents as possible. This search protocol is outlined in this SLR’s sections 3.1, 3.2, and 3.3 and is aimed to reduce individual bias, so others who follow the same protocol would achieve the same results. A relevant study may be misclassified into a removal group during the selection process and vice versa. To reduce the bias in selection of papers, we defined the review protocol with clear inclusion and exclusion criteria for each selection phases. Although the protocol was not reviewed by an independent reviewer, which would have added rigor to the review process, one of the co-authors has extensive experience in conducting secondary studies, which could reduce procedural risks. Prior to applying the inclusion and exclusion criteria, the two reviewers conducted a pilot review, as outlined in section 3.3 of this SLR.

In the pursuit of a comprehensive SLR, it is imperative to acknowledge and address potential sources of bias inherent within the studies themselves that comprise the SLR. The integrity of our synthesis is inherently contingent upon the methodological rigor and quality of the individual studies under review. Therefore, we recognize that the robustness of our findings cannot surpass the caliber of the selected primary studies. In our evaluation of the studies included in this SLR, a primary concern is the potential for bias to influence the outcomes and conclusions presented, due to the inclusion of studies with deficiencies in study design, participant selection, data collection and/or analytical procedures. Given that 81 of the 91 sources are from peer-reviewed journals and that the other sources also went through peer-review prior to publication, we believe our synthesis represents the overall quality of the studies selected.

## 5. Discussion

*Results of the search and limitations*

This section presents our answers to the three RQs in Section 5.1 (RQ1), Section 5.2 (RQ2), Section 5.3 (RQ3), as well as the limitations of this SLR.

### 5.1 RQ1: What are the characteristics of migrant entrepreneurs investigated in primary studies, in the European context?

*5.1.1 Characteristics of migrant entrepreneurs in Europe*

When discussing the characteristics of migrant entrepreneurs in Europe, it makes sense to revisit the definitions from the beginning of this SLR. We view migrant entrepreneurs as foreign born, following the definition by Dheer (2018), who writes they can “identify, create and exploit economic opportunities to start new ventures in their destination nations” (p. 558). We also mentioned that some of the literature uses the term immigrant entrepreneur while other literature uses the term migrant entrepreneur and that both terms are relevant to this SLR. A large number of studies included in our SLR did not explicitly share a definition of entrepreneur or accompanying adjectives such as ‘migrant’ or ‘immigrant’. Several sources in our SLR did not use the terms migrant entrepreneur or immigrant entrepreneur. They instead either used the term diaspora entrepreneur, ethnic entrepreneur, refugee entrepreneur, or referred to entrepreneurs by their home country. It was often the case that sources with a broader coverage of migrant entrepreneurship or immigrant entrepreneurship did not provide an explicit definition of these terms, despite using them as a focus in their studies. The sources that were most explicit with their definitions are listed on Table 6. Some of the definitions clash with ours, because we do not include second generation immigrants, who are born in the host country, as migrant entrepreneurs. Moreover, “country of origin,” as used by Širec and Tominc (2017), could be subject to interpretation. We also note that most of the 91 sources do not include explicit definitions on migrant or immigrant entrepreneurship. Table 6 displays an overview of the definitions. It is possible to categorize the characteristics revealed in the studies, into those that are unique to migrant entrepreneurs and those that apply to all entrepreneurs. Frequently studied characteristics that apply to all entrepreneurs include formal education, prior vocational experience, sector of business activity, gender, age, nationality, reasons for becoming an entrepreneur, marital status, and parental status. 10 of the studies that focus on migrant background investigate differences between first- and second-generation migrants and sometimes even into the third generation. This is not within the scope of this literature review, which only is concerned with first-generation migrants.

**Table 6.** Definitions of immigrant entrepreneur and migrant entrepreneur in the literature of the SLR.

Term used	Definition	Source
Diaspora entrepreneur	‘Forever’ immigrants settled in a country other than their country of origin, who have a cultural understanding of both their host and home country and engage in business.	Osaghae and Cooney (2020)
Ethnic entrepreneur	Individuals with similar national backgrounds or migration experiences who establish a business in their new host country. Thandi and Dini (2010) blend the term with immigrant entrepreneur, referring to immigrant/ethnic throughout the article. Self-employed migrants.	Birdthistle (2019); Lazaridis and Koumandraki (2003); Thandi and Dini (2010)
Immigrant entrepreneur	Business owner/founder born outside the host country. Some authors stress non-Western origin. Some studies only focus on new technology-based firms or food business. Some studies include both first and second generation.	Abbasian and Yazdanfar (2015), Bolzani and Boari (2018), Glinka and Hensel (2020), Murphy et al. (2020), Širec and Tominc (2017), Yazdanfar et al. (2015)
Migrant entrepreneur	A person who moves to another country and establishes a business; born abroad or parents board abroad and establish/manage new venture. Szczygiel qualifies with at least two years residency in the host country.	Berntsen et al. (2021), Solano (2021), Szczygiel et al. (2020)
New migrant entrepreneurs	Arrived at host country within last 25 years who aim to succeed at business.	Hagos et al. (2019)
Refugee entrepreneur	Authors imply forced migration/fleeing dangerous conditions and seeking to establish/establishing a business in the host country; they mention home-country conditions that impede building transnational business contacts compared with other migrant entrepreneurs.	Barth and Zalkat (2021); Embiricos (2020); A. Harima, Freudenberg, and Halberstadt (2020); Hartmann and Schilling (2018); Johnson and Shaw (2019); Mawson and Kasem (2019); A. D. Meister and R. Mauer (2019); Nijhoff (2021); Qin (2021)

Previous entrepreneurship literature follows the line of thought from Bourdieu (1984) by looking at the impacts of forms of capital on the establishment and maintenance of a business. Inspired by the cultural, economic, social, and symbolic capital of Bourdieu (1984), the studies covered in this SLR show a shift into three distinct forms of capital that frequently appear often in assessment of starting and running a business: financial, human, and social capital ( Baklanov *et al.*, 2014; Eroğlu, 2018; Grubbström and Joosse, 2021) . The concept of social capital is also inspired by Granovetter (1973) whose seminal work, *The Strength of Weak Ties*, researches how bridges across social networks relate to social mobility and social cohesion. Basit (2017) also cites Granovetter (1985) and Uzzi (1997, 1999), to share how social embeddedness is a framework by which to study economic activity, from which researchers investigate how social relations shape entrepreneurial activities and outcomes. Several sources in the SLR stress the importance of support mechanisms that build up both weak and strong ties, including Bouk, Vedder and Poel (2013), Harima *et al.* (2020), and Noor (2021). Waldinger (1995) also played an important role in earlier literature on social capital in migrant entrepreneurship, with his study on the construction industry in New York City, and is cited sources in the SLR by Bagwell (2008) and Barth and Zalkat (2020). This SLR uses the categories of financial, human, and social capital to classify the characteristics gathered from the studies. The literature stream considers primarily the *positive* impacts of the social capital from one’s ethnic group, as we did not encounter many pitfalls mentioned by Portes and Landolt (2000). However, the study by Andersson (2021), which is included in this SLR, does mention that entrepreneurship by new arrivals can depend on the extent of entrepreneurial engagement by co-ethnics already in the host-country. Andersson (2012) and another study included in the SLR, by García, Molina, and Lubbers (2014) also mention that ethnic enclaves can slow down the integration process and new arrivals’ ability to learn the host country’s culture and language.

For the sake of this study, emphasis is on the characteristics specific to migrant entrepreneurs rather than entrepreneurs in general. Table 7 shows the key factors studied. It is worth noting that the social capital factors are those that are the most specific to migrant entrepreneurs. The implication is that it is important for migrants to learn the local language and establish social capital by learning the local culture and by building up a local network, to establish and operate a business in the host country. It is also evident that it is more difficult for migrants to move to the host country and immediately start a business unless they already have business experience or relevant experience from their home countries ( Barth and Zalkat, 2021). The more time a migrant spends in the host country, the easier it appears to establish and run a business in the host country.

**Table 7.** Characteristics of migrant entrepreneurship.

Category	Factor	Description	Reference
**Human Capital**	Work experience in the home country	Work experience from the migrant’s country of origin.	S3, S27, S86
	Work experience and qualifications in the host country	Work experience and professional credentials from the host country.	S27, S72
	Length of time in host country	How long has the migrant lived in the host country?	S3, S4, S6, S26, S33, S34, S35, S36, S48, S53, S55, S63, S67, S74, S75, S81, S86
	Local language competence	What level of ability does the migrant have in the host country language?	S1, S3, S28, S47, S75, S88
**Social Capital**	Social capital/social situation /network participation/role of home country conditions/age on leaving home country/income in home country before leaving	Various authors report the network and social capital, which either way help entrepreneurs to connect to the ecosystem and the market, while historical income in the home country sets the foundation for social status in the host country	S17, S27, S44, S47, S52, S61, S71, S76, S81
	Cultural skills	Understanding the host culture helps build the foundations for integration.	S28, S47
	Legal/residency status in host country/citizenship status	Legal status is the first step to building social capital in the host country, includes international protection status.	S10, S30, S33, S49, S84, S86, S88
	Residence route	Previous residence* /migration route to host country* /passing through transition country to host country*	S37, S53, S58
	Individual or joint migration	Entered host country alone or with relative/ other/or to join family already in the host country /married to a citizen of the host country/was male ancestor a migrant	S10, S47, S53 S57, S58, S68, S79
	Motivations for relocation	Why did the migrant move to the host country? This includes ‘lifestyle’ migration, forced migration, and whether the stay is temporary or permanent.	S13, S17, S30, S36, S52, S58, S68, S72
	Ethnic identity or enclave	The ethnic group identity can be strong or weak, the community size big or small, and its resources vast or lacking.	S11, S28, S44
	Location in host country	Some studies are specific to a geographical area in the country or compare different parts of the same country. Location will have implications on building up social capital with the local community as well as co-ethnics in the community.	S35, S38, S40, S49, S72, S74, S75, S76, S91

*5.1.2 Human capital*

As mentioned in the background of this article, migrant entrepreneurs are people who have moved to a different country and have started businesses there, and they have an array of backgrounds and offer a unique set of characteristics, experiences, and skills to run their businesses.

Table 7 shows human capital factors that differentiate migrant entrepreneurs from native entrepreneurs. One of these factors is whether migrant entrepreneurs obtain their work experience in the home country or the host country. Mixed-country work experience can provide migrant entrepreneurs with unique perspectives on how to conduct business in cultural and economic environments and offer advantages by helping bridge gaps between the home country and the host country ( Bolzani and Boari, 2018; Van Delft *et al.,* 2000). In this respect migrant entrepreneurs have some advantages over native entrepreneurs, provided that they are embedded with the cultural norms and social networks of both the home and host country ( Bolzani and Boari, 2018; Meister and Mauer, 2019). As with work experience in the host country, qualifications from the host country are also an important consideration. When a migrant entrepreneur has obtained educational qualifications and certifiable skillsets in the host country, such credentials not only help establish individuals as experts in their fields, but when the credentials are from the host country, it demonstrates a higher level of local embeddedness which means a larger local network and greater understanding of the local bureaucracy and regulations, which in turn can ease the processes of starting and running a business in the host country. Along these lines, the length of time in the host country and ability in the host country language are all key characteristics which contribute to this local embeddedness and higher level of local understanding, and which also appear on Table 7. Examples include a deeper understanding of the local market ( Szczygiel *et al.,* 2020) and a greater likelihood of established relationships with key players in the community. On the other hand, those who are newer to the country may have a fresh perspective and may be more open to trying new things. Whereas local language competence eases communications with customers and clients and may be more successful in building relationships within the community ( Nijhoff, 2021). It also means that migrant entrepreneurs will be able to compete on a service level rather than merely on price ( Valenzuela-García *et al.,* 2014).

*5.1.3 Social capital*

Some migrants arrive in their host country with more social capital than others. As mentioned in the background section of this article, there are many reasons why people move to another country. Of the eight articles that explore this area further, the motivations for migration include one investigation of “lifestyle migrants” who have chosen to immigrate to a new country not primarily for economic reasons, but rather for personal or lifestyle reasons ( Munkejord, 2017a). These entrepreneurs may be drawn to the country for its culture, climate, or other non-economic factors, and may be more interested in starting businesses that reflect their personal interests and passions. Seventeen studies focus on refugee entrepreneurship, where the motivation to move to another country is to flee dangerous conditions in the host country. This is also known as forced migration. The forced migration studies by Qin (2021) and Harima and Freudenberg (2020) are listed on Table 7 for their contributions. Harima *et al.,* (2020) add that while entrepreneurship can be a path for the vocational integration of refugees, they need special support beyond what native entrepreneurs would require. Along with motivations for migration, it follows that scholars are interested in knowing whether people were joining family in the host country, entering the host country with others, or traveling alone.

An important part of building social capital is having legal status in the host country, with citizenship status being the highest level of inclusion. Legal status also frequently appears in the literature. When migrant entrepreneurs lack legal status, it means they are engaging in the underground economy with implications of missed tax revenue for the host country and missing social welfare benefits and protections for the migrant entrepreneurs. When considering legal status, it is also the case that some countries do not allow asylum seekers or refugees to work or engage in entrepreneurship until certain conditions are met ( Lintner and Elsen, 2020). This can have severe implications on the integration process, especially if the migrants are kept apart from the host society in isolated processing centers or shelters for extended periods of time. Another study focuses on the European Union (EU) movement of people which makes it easy for EU citizens to gain legal status in other EU member states ( Bruzelius, 2020).

A final social capital factor to note is the location in the host country. Some of the studies explore comparative relationships between different locations within the same host country. In some host country communities, a town or city may have a strong ethnic enclave in place ( Andersson, 2021). Results show that ethnic enclaves frequently play a role on migrant entrepreneurship activities. For example, there may be an inclination to imitate other co-ethnics who appear to be successful entrepreneurs in a specific industry ( Andersson, 2021). On one hand, co-ethnics may be able to help introduce and explain the host culture, society, and regulations to new arrivals and opportunities exist for migrant entrepreneurs to leverage the co-ethnic network to access the market of their co-ethnics ( Van Delft *et al.,* 2000). On the other hand, ethnic enclaves can also have an effect of slowing the integration process by hindering the speed at which new arrivals learn the local language and have contact with native society ( Andersson, 2021; Valenzuela-García *et al.,* 2014).

### 5.2 RQ2: What do we know about challenges that migrants face as entrepreneurs in European host countries?

Several challenges discussed in the literature are not specific to migrant entrepreneurs; these include issues with private life, gender discrimination, competition, marketing issues, and problems with advisors and lack of them, as well as a range of insufficiencies, including professional experience, skills and training, access to resources such as personnel, real estate, and financing. Financing, however, can be more central of an issue to migrant entrepreneurs than to native entrepreneurs, due to higher unemployment rates, a lack of access to bank loans, greater financial uncertainty, and difficulty in receiving funding from mainstream banks. As pertaining specifically to migrant entrepreneurs, Table 8 highlights the main points from the literature. The most frequently mentioned challenges specific to migrant entrepreneurs include discrimination and a lack of the following: cultural and social understanding, local network, proficiency in the local language, understanding local laws and regulations, and legal status in the host country.

**Table 8.** Challenges for migrant entrepreneurs.

Category	Factor	Description	Reference
**Social Capital**	Lack of resources	This can be social resources, on the individual level or the ethnic group level.	S20, S28, S34, S46, S52, S64
	Lack of network	Lack of local network, social support and local business connections in host country hinders progress of entrepreneurial endeavor. This can also include lack of trust and inability to attract talent.	S1, S4, S5, S7, S23, S27, S29, S33, S34, S44, S48, S50, S63, S73, S76, S85, S87, S90
	Ethnic enclaves	Ethnic enclaves can make for slower integration/learning about host country language and culture.	S6, S62
	Legal Status: Political and institutional issues relating to status in the host country	Issues such as residency status, citizenship status, legal status in the host country, which can restrict ability as entrepreneur. This includes refugee status/asylum status and lack of support for those with such status and issues with becoming documented in the host country.	S1, S2, S4, S7, S8, S10, S19, S28, S32, S48, S58
	Lack of cultural and social understanding	Cultural and social differences can create challenges for migrants. This can include a lack of business knowledge in the host country and difficulties integrating into the host country, which can be exacerbated by the ethnic enclave.	S7, S13, S16, S17, S23, S25, S30, S34, S35, S45, S46, S48, S52, S57, S60 S62, S64, S58, S73, S75, S78, S81
	Discrimination of outsiders	Local populations may have unconscious bias, discrimination, fear, or racism towards migrants. It can be difficult for immigrants to build trust with the community. Policies can exclude migrants.	S7, S10, S13, S23, S25, S26, S34, S35, S43, S49, S56, S58, S64, S72, S75, S84, S86, S91
	Perpetuating Stereotypes	The very construction of ‘migrant entrepreneurship’ creates an otherness and the comparison to the local/native entrepreneur, which can perpetuate stereotypes. Immigrants can be lumped together in one group despite their uniqueness and individual differences. It can also create an in-group/out-group scenario.	S7, S54, S78
	Lack of information	Info gap means missing out on aspects that are relevant to starting and running the business as well as tender offers. This may be due to lack of local network to find out the information needed.	S10, S34, S76, S80, S90
**Financial Capital**	Lack of resources	This can be financial resources (with implications on difficulty to afford housing as well) on the individual level or the ethnic group level.	S20, S24, S28, S34, S46, S52, S64
	Unemployment	Migrants face higher unemployment rates than the native population. They also are more prone to blocked mobility.	S26, S39, S42 S71, S75, S82, S84, S85, S91
**Human Capital**	Lack of resources	This can be human capital resources, on the individual level or the ethnic group level.	S20, S28, S34, S46, S52, S64
	Lack of language proficiency in the host country language	Limited ability in the host country language impacts migrants’ abilities to communicate and understand. It can also mean they need to compete on price. It can also be difficult for migrant entrepreneurs to have access to language classes that suit their schedules.	S3, S7, S9, S10, S23, S24, S25, S26, S27, S34, S36, S42, S43, S48, S55, S58, S72, S78, S81, S85, S88
	Lack of understanding of local business laws, regulations, and taxes	Bureaucracy can be complex and especially difficult for migrants to understand. This can include lack of institutional knowledge or support.	S1, S3, S12, S13. S16, S17, S20, S27, S28, S34, S36, S48, S55, S59, S66, S68, S82, S83, S85
	Lack of credentials	Migrants lack qualifications and credentials or recognition of them, that can allow them to become entrepreneurs	S3, S9, S23, S26, S37, S43, S49
**External Factors**	Integration programs	Integration programs that encourage migrants to find a job instead being allowed to become an entrepreneur under refugee status	S4, S24, S25

*5.2.1 Social capital*

Table 8 highlights the social capital factors that are challenges migrants face as entrepreneurs in European host countries. Here, lack of cultural understanding frequently appears as a challenge. This challenge can arise in several contexts, whether from the lack of understanding refugee resettlement policies in the host country ( Qin, 2021), the creation of in-group/out-groups ( Rashid and Cepeda-García, 2021), or a general disadvantage in establishing an enterprise in the host country ( Bolzani and Boari, 2018). In addition, Meister and Mauer (2019) and Qin (2021) postulate that refugee entrepreneurs face a greater lack of social capital than other migrant entrepreneurs, which would require a specific support model for them, which is the case for the Fresh Start Programme, which also finds that there is not a one-size-fits-all approach for migrant entrepreneurship in various settings within Europe ( Wade, 2020). Discrimination by the host community is another frequently mentioned challenge that migrant entrepreneurs face; this results in missed opportunities ( Solano, 2021). Initiatives from civil society to increase contact between native and migrant populations can help build cross-cultural familiarity and reduce discrimination ( Embiricos, 2020). Meister and Mauer (2019) focus on the refugee context and point out that discrimination creates a lack of trust that is a part of an overall negative societal perception of refugees, with the greater implications being limited socio-economic involvement and lack of engagement in the legal-institutional environment of the host country. Lack of network with natives in the host country is also frequently mentioned. Van Delft *et al., (2000)* note that this can lead to migrant entrepreneurs’ exclusion from the mainstream economic activity of the host country.

*5.2.2 Human capital*

Challenges in the area of human capital are also frequently mentioned in the literature. Lack of proficiency in the language of the host country is the most frequently mentioned issue. This has implications such as communicating with customers, suppliers, submitting tenders, ability to negotiate, and ability to comply with local laws, regulations, and tax regimes. Such issues are also frequently mentioned in the literature as a challenge for migrant entrepreneurs. In addition, lack of credentials can prevent entrepreneurs from being able to operate in areas of their expertise if their qualifications are not recognized in the host country. Lack of resources can fall into the human capital arena, either on an individual or ethnic group level. In other cases, the issue can fall under financial capital, as mentioned in the next paragraph.

*5.2.3 Financial capital*

Lack of resources in the financial capital area include lack of money, difficulty to afford housing, and lack of access to financing. Migrants face higher unemployment rates than the native population ( Evansluong and Ramírez-Pasillas, 2019; Szczygiel *et al.,* 2020) which can lead to a lack of financial capital. Barth and Zalkat (2020) point out a lack of financial expertise is also an issue for many migrant entrepreneurs. Financial training, while falling under the human capital area in the previous section, is an important consideration for policy makers and practitioners when looking at funding a new migrant entrepreneurship venture. Meister and Mauer (2019) emphasize that forced migrants are likely to face a lack of financial capital due to the nature of suddenly fleeing from their home country. In a study of migrant entrepreneurship support programs across six European cities, Van Delft *et al.,* (2000) find that despite the financial challenges that migrant entrepreneurs face, programs that emphasize education and training appear to be the most successful, which reinforces the view that financial support should come after ensuring that human capital reaches a sufficient level for migrants to establish and run a business.

*5.2.4 External factors*

External factors are an issue when a migrant has refugee status, because of specific constraints such as rules that do not allow refugees to become entrepreneurs. If integration programs direct migrants to find a job and do not consider the individual desires and motivations of the migrants, who may be interested in entrepreneurship, then this can be a challenge that hinders entrepreneurship ( de Lange *et al.,* 2021; Harima and Freudenberg, 2020; Wade, 2020).

### 5.3 RQ3: What do we know about reported policies as support mechanisms for migrant entrepreneurship in the European context?

Financial capital initiatives include bank loans, donations, subsidies, and tax relief, with general access to finance, funding, or financial support most often mentioned in the literature. Human capital initiatives include programs that will help aspiring migrant entrepreneurs pass the necessary training for required certifications to operate in their chosen industry, entrepreneurial and language training, co-development between small migrant enterprises and larger companies, and specific support for internationalization. Social capital initiatives build cultural understanding, network, and provide helpful information about entrepreneurial support that is available locally and nationally. Table 9 shows that entrepreneurial training, language training and mentoring are the main activities as human capital support; the table also includes a number of external factors which are “opportunity structures” divided into market, regulatory, and state categories ( Berntsen *et al.,* 2021).

**Table 9.** Migrant entrepreneurship support initiatives and policies.

Category	Factor	Description	Reference
**Social Capital**	Includes building up strong and weak ties	Providing information is a part of building up the social capital and network. This can also be information to help migrant entrepreneurs understand the support structures themselves. Build network /Access to social networks /tapping into social capital/building up non-ethnic network/access to accountants and lawyers /contact with suppliers /support communities/networks /forming co-operatives/knowledge exchange	S1, S2, S4, S9, S10, S13, S14, S15, S16, S17, S19, S21, S27 S33, S34, S50, S53, S54, S64, S67, S71, S73 S78, S80, S85, S86
	Cultural training	Can include mentors from host country to help explain local culture and information about host society/focusing on cultural differences	S13, S34, S37, S81
	Emotional support	Emotional support and encouragement/structured network of psychological support to assist migrant entrepreneurs to feel more ‘at home’. Migrants often turn to the ethnic group and to family for emotional support as well.	S17, S20, S26, S35, S47, S53
**Social Capital from the Ethnic Group**	Ethnic enclave creation fosters learning from people from the same country who can help access the local ethnic market: especially if the ethnic enclave has a high share of self-employment, new arrivals will be more likely to enter entrepreneurship.	Consider enclave resources/resources from the ethnic group (includes info, opportunities, loans, and access to ethnic labor) /funding from co-ethnics	S6, S21, S28, S51, S54. S68, S73
**Financial Capital**	Finance	Access to finance/to financial support/funding/capital	S4, S10, S13, S14, S15, S16, S26, S36, S53, S54, S64, S70, S73, S77, S83, S86
**Human Capital**	Mentoring/advising	Providing mentors / advisors /learning exchange/knowledge building /coaching-mentoring/advisory services & business support/mentorship from success refugee entrepreneurs/individual counselling	S5, S9, S10, S12, S13, S15, S20, S27, S36, S37, S48, S53 S70, S71” S73 S80, S82, S83, S86, S87
	Language training	Teaching migrants the host country language: reading, writing, speaking, and listening skills.	S13, S33, S34, S37, S81
	Entrepreneurial Education/Training	Understanding of legal and institutional aspects building niche markets, product-market fit, skill building, up-skilling, training on financial and marketing plans, personal development, market evaluation, tailored support, market access	S8, S9, S10, S13, S15, S17, S20, S25, S27, S33, S34, S36, S46, S48, S52, S54, S59, S67, S71, S73, S78, S85
**External Factors**	Market	Examples include digital platforms and new technology that helps people become entrepreneurs and access new markets.	S11, S22
	Regulation	Examples include childcare for women entrepreneurs and educating banks to help migrant entrepreneurs	S9, S49, S53, S67, S72
	State: Civil society	Government funds civil society organizations that help migrants.	S7
	State: Immigration policy	Entrepreneurship visa, allowing refugees to enter entrepreneurship and employment, EU free movement policy, connecting immigrants with entrepreneurship support opportunities. Immigration policy that connects migrants with entrepreneurship from the start.	S4, S12, S14, S23, S30, S42, S43, S45, S48, S55, S66, S89
	State: Differentiated assimilation policies	This entails an individual level of support policies because some target groups require specific support to cater to their needs since they encounter different integration.	S32, S43, S66

External factors broken down into three “opportunity structures”: 1) market, 2) regulation, and 3) state. Source: Berntsen *et al.,* (2021), page 272.

*5.3.1 Human capital*

Table 9 displays various human capital factors to consider for migrant entrepreneurship support initiatives and policies. Education and training, followed by mentorship and advice are the most commonly cited areas of support, followed by language training. The literature shows evidence that to establish and run a business in the host country, migrant entrepreneurs require adequate education and a relevant skillset to navigate the complexities of the local bureaucracy, tax rules, legal issues, and accountancy, in addition to the marketing know-how, resource management and leadership skills required to run a business. Research by Nijhoff (2021) in the Netherlands demonstrates that the issues of language and local bureaucratic complexities should be the first foundations to cover for migrant entrepreneurship support programs. Backman *et al.,* (2021) also place language learning as a priority, in addition to cultural training to learn about the host country society. Beyond these foundations, relevant education and training can help to bring aspiring migrant entrepreneurs to a level of self-sufficiency to help them manage their businesses. Mentorship and business advice are also helpful to fill in knowledge gaps and to answer questions for specific areas where migrant entrepreneurs may need extra help. Although we see evidence that language learning is a cornerstone component in the journey of a migrant entrepreneur, it is less mentioned as a support area in the literature that is covered in the present SLR, and instead appears most often as a characteristic that is required to run a business in a new country. Wade (2020) emphasizes the need for language support and intercultural training to be a part of entrepreneurial support programs that are specific for migrant entrepreneurs, such as the case with the Fresh Start Programme.

*5.3.2 Social capital*

In Table 9, there are also social capital factors to consider for migrant entrepreneurship support initiatives and policy, with lack of local networks being a frequently mentioned factor. To set up and operate a new venture in the host country, migrant entrepreneurs will benefit from a local network comprised of both weak and strong ties ( Bouk, Vedder, and Poel, 2013). One way that migrant entrepreneurs can build up both weak and strong ties is by joining a local accelerator ( Noor, 2021), with Harima *et al.,* (2020) and Meister and Mauer (2019) providing extra emphasis on business incubators for refugee entrepreneurs that explicitly incorporate the need for understanding and tapping into social capital in the host country. Bouk *et al.,* (2013) examine the differences between strong and weak ties for migrant entrepreneurs and find that strong ties provide the key resources during the startup phase for those who have a network of entrepreneurs already in the host country. When migrant entrepreneurs’ business network in the host country is missing, they find that migrant entrepreneurs should build up weak ties to help them in the initial phases of their business journey. Research by Colombelli *et al.,* (2021) shows that successful entrepreneurs as role models who are the same gender as the individual just starting out are also important. Therefore, entrepreneurial support programs should take this into consideration. Entrepreneurial support programs should also consider whether the migrant entrepreneur is married to a local of the host country, since there is an existing set of weak and strong ties from the local network of the spouse ( Munkejord, 2017b). Furthermore, research by Mason (2008) demonstrates the need for entrepreneurship support initiatives to be bottom-up, that is to say, originating from the migrants themselves, because the research shows that top-down initiatives do not help migrant entrepreneurs create a viable business within a reasonable time frame, while Lyon *et al.,* (2007) shows that entrepreneurship support should consider the individual social context of migrant entrepreneurs as well, since this leads to different qualities of entrepreneurship within a local host economy, regardless of ethnicity. Additionally, research by Van Delft *et al.,* (2000) leads to the concern that migrant entrepreneurship support’s emphasis is on exploiting economic potential rather than focusing on the problems that migrants face when entering labor markets in their host countries. In this respect, it is important to consider other factors of social capital that appear in this SLR, such as training about the local culture and society (Johnson and Shaw, 2019; Qin, 2021) as well as providing emotional support ( Hagos *et al.,* 2019; A. Harima, Freudenberg, and Halberstadt, 2020).

*5.3.3 Financial capital*

Financial capital is another part of migrant entrepreneurship support initiatives which frequently appears in the literature. Research by Kontos (2003) shows that a specific support policy in Germany, known as a ‘bridging allowance scheme’ to finance the stabilization phase of a new business, appears to be of greatest benefit to female migrant entrepreneurs, who have been socialized toward self-employment.

*5.3.4 External factors*

Berntsen *et al.,* (2021) suggest that we can break external factors into three “opportunity structures”: 1) market, 2) regulation, and 3) state. Of these three opportunity structures, those taking place at the state level appear most frequently in the literature, in particular concerning immigration and differentiated assimilation policies. Initiatives such as entrepreneurship visas for qualifying individuals ( Noor, 2021) and the European Union’s free movement policies ( Bruzelius, 2020) are examples of immigration policies that help qualifying migrant entrepreneurs to establish and run a business in the host country. As for market opportunity structures, a couple of articles provide examples of how new digital platforms have helped migrant entrepreneurs to access customers and formulate pricing models ( Wang and Chen, 2021; Webster and Zhang, 2020). Examples of regulation opportunity structures include childcare services for women entrepreneurs ( Bashir, 2018; Ozasir-Kacar and Essers, 2021) and educating banks to help migrant entrepreneurs with financing ( Piperopoulos, 2010).

## 6. Limitations

SLRs offer valuable insights into research domains, but they are not without limitations. One significant challenge inherent is the potential for overlooking relevant studies. This is a salient issue with this SLR because it bridges two disciplines, entrepreneurship and international migration, which in themselves span diverse disciplines. International migration spans disciplines such as demography, economics, geography, history and sociology ( Inglis, Khadria, and Li (2019), and entrepreneurship, spans disciplines in economics, management, psychology, and sociology ( Davidsson, 2009). To address this, a comprehensive search protocol was implemented to retrieve a wide range of documents, outlined in sections 3.1, 3.2, and 3.3 of this SLR, aiming to minimize individual bias. However, the risk remains that a pertinent study might be erroneously categorized during the selection process, affecting the review's comprehensiveness. Indeed, during the open review process, we received feedback that the reviewer was aware of an article that met our search criteria but still did not appear in the SLR. Efforts to mitigate this bias include clear inclusion and exclusion criteria defined in the review protocol for each selection phase. While an independent review of the protocol would have bolstered the review's rigor, the involvement of an experienced co-author with a background in secondary studies helped counterbalance procedural risks. The piloting of the review process by the authors further contributed to refining the selection methodology.

An additional limitation of this study is that it does not account for the impact factor of the journals included, which is a measure that the systematic review by Aliaga-Isla and Rialp (2013) achieved. Nor does this study engage in citation analysis to focus on the most impactful articles, which is a measure taken in the systematic review by Sithas and Surangi (2021).

The integrity of the synthesis conducted within this SLR is contingent upon the methodological rigor and quality of the individual studies included. The authors acknowledge that the robustness of the findings from this SLR cannot exceed the caliber of the selected primary studies or analytical procedures. Despite the inclusion of primarily peer-reviewed journals (81 out of 91) and ten remaining peer-reviewed studies, it is important to recognize that the SLR’s conclusions are tethered to the overall quality of the selected studies. In terms of synthesis, efforts were made to categorize and analyze findings systematically. While the process of extracting information and categorizing studies using original terms and predefined criteria is meticulous, it may still be influenced by researchers' interpretation. The adoption of tailored thematic analysis with open coding adds a degree of subjectivity to data extraction, potentially impacting the objectivity of results. Although systematic working sessions between co-authors were conducted to enhance consistency during data extraction and synthesis, individual biases might subtly influence the process. Collaborative iterations during the synthesis process helped maintain focus on the research questions and ensure a balanced approach where two people working together during the iterations helps reduce the bias of a single person. However, despite these efforts, the inherent subjectivity in identifying relevant paragraphs and texts could introduce unintended bias into the analysis. While systematic literature reviews offer valuable insights, they are susceptible to limitations such as potential oversight of relevant studies, bias in data extraction and analysis, and the dependence on the quality of selected primary studies. The authors aimed to mitigate these limitations through systematic procedures, collaboration, and transparent reporting to enhance the validity of the SLR's findings. However, it is of merit to acknowledge the inherent challenges of the methodology.

In addition, Table 11, which lists adopted theories of the 91 studies included in this SLR, does not list every implicit theory that the studies incorporated into their research, nor does it provide an in-depth analysis of how the theories listed are applied. Such an in-depth analysis could be worthy of future research because it would provide an overview of how scholars are applying theory and how theory application relates to research methodologies and the unit of analysis for the studies reviewed.

## 7. Implications for Practice, Policy, and Future Research

In terms of support for migrant entrepreneurs, our review attempts to cut through the complexity of the literature to demonstrate that migrants need to build the foundations for human and social capital in the host country prior to focusing on financial capital and entrepreneurship. This has implications for host country governments and practitioners managing migrant entrepreneurship support programs, since without first having a solid level of human and social capital in the host country, migrant entrepreneurs may be doomed to either failure or low margin businesses (and possibly illegal or non-registered businesses) with long working hours and sub-par conditions ( Bashir, 2018; Solano, 2021; Van Delft *et al.,* 2000), rather than contributing strongly to the economies of their host countries. This would indicate that offering training opportunities related to learning the local culture and language, educational advancement, networking, and volunteering should be considered higher priority than providing financial support, except in cases where migrants first demonstrate enough human and social capital to serve as the foundations for a future as entrepreneurs in their host country.

As noted in the categorization of the data, the results of this study also reveal three key thematic clusters as emerging from the 91 sources included in this review. These are namely: financial capital, human capital, and social capital. The financial capital cluster includes categories such as access to financing, employment status when starting a business, personal wealth, and family wealth. The human capital cluster includes categories such as: previous experience, professional background, education, and skillsets. The social capital cluster includes categories such as: host country experience, local credentials, language competency, local network, length of time in host country and legal residency or citizenship status in the host country. These three clusters serve to understand both the characteristics of migrant entrepreneurs as well as the challenges they face when financial, human, and social capital are missing.

### 7.1 A conceptual framework of migrant entrepreneurship support

**Figure 4.**
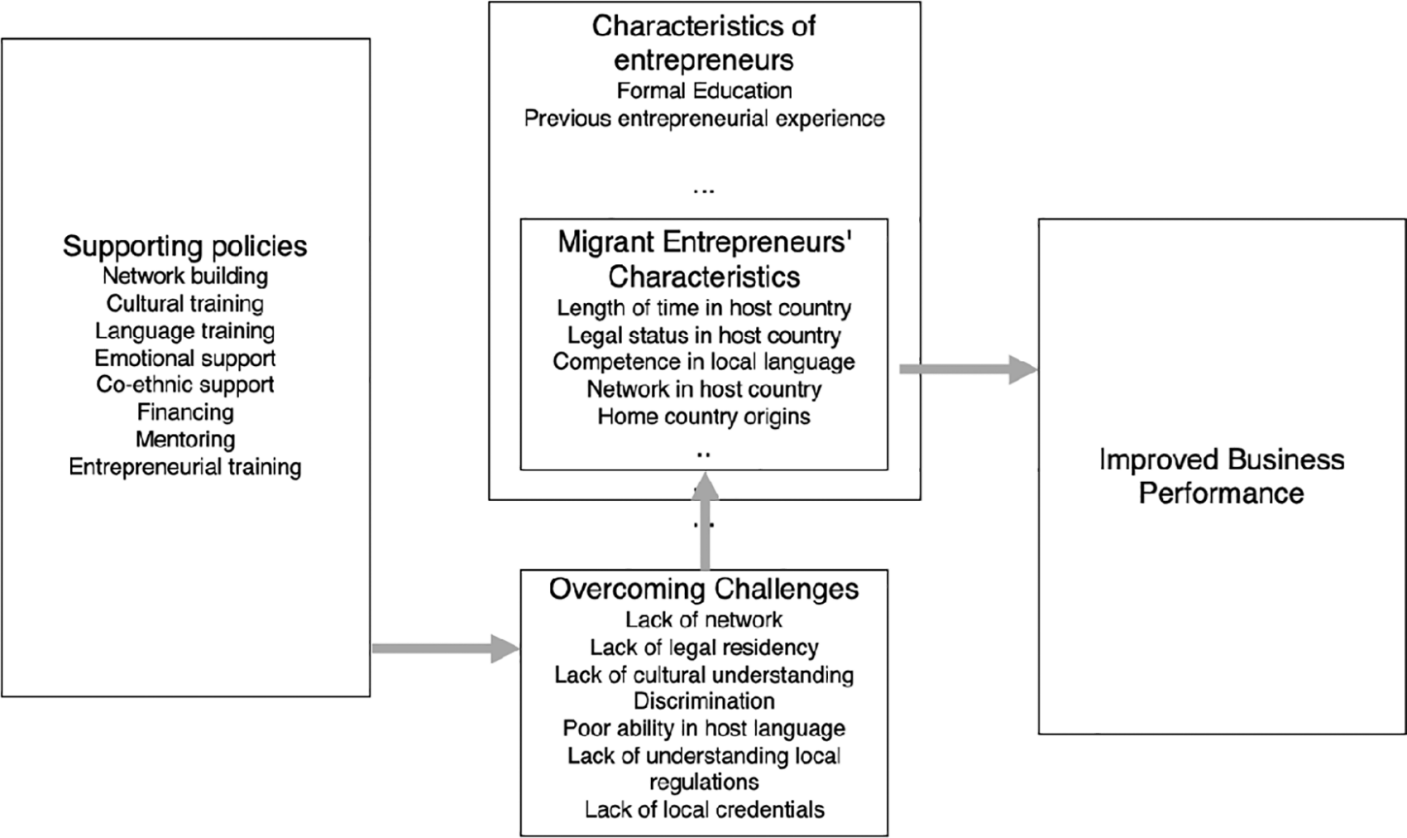
Conceptual framework that reflects the findings from the research questions.

Figure 4 displays some of the key reflections from the findings relating to our research questions.

On the left of the figure, supporting policies encompass key areas that migrant entrepreneurs require to establish, maintain, and grow a business in the host countries. These support areas cover the range of financial, human, and social capital. Evidence shows that cultural and language training are the first foundations necessary prior to building up the remaining support areas. The support then helps migrant entrepreneurs to face the challenges that the literature emphasizes, starting with cultural and language understanding and then tackling other areas such as acquiring local credentials, understanding local regulations, and building up a local network. Consideration of the migrant entrepreneurs’ previous professional and entrepreneurial experience and educational experience are key when considering what kind of entrepreneurial support to offer. Two key issues that also need to be considered are discrimination and legal status, which along with a migrant entrepreneur’s home country origin, have an impact on the path forward as migrant entrepreneurs. To this extent it is important to individualize the entrepreneurial support offered rather than lumping migrant entrepreneurs into one single group. This may help avoid an in-group/out-group scenario and may help overcome issues discrimination. The idea is that entrepreneurial support will then assist migrant entrepreneurs to establish and run a business that will help them become economically independent and contribute to the economies and societies of their host countries.

### 7.2 Implications for practitioners

For those running entrepreneurship support programs, it is important to include clear goals for the programs and to include metrics to evaluate their effectiveness according to the desired goals of the program. A takeaway from this SLR is that improved business performance would be a logical desired outcome for entrepreneurship program participants. So setting up ways to track improved business performance in terms of number of employees and profits are examples of metrics that can accomplish that. Other considerations are the catalysts that help generate improved business performance. Indications are that transnational entrepreneurship, defined as business taking place across national borders ( Drori, Honig, and Wright, 2009) would be worth instilling into entrepreneurship support program participants long-term plans because transnational enterprises are more profitable than other forms of migrant entrepreneurship ( Solano, 2016). The IntEnt non-profit incubator investigated in the Riddle, Hrivnak, Nielsen (2010) had the uniqueness of being dedicated to transnational diaspora entrepreneurship. Wang and Liu (2015) found that transnational firms have higher payroll per employee than other firms, regardless of whether the other firms are migrant or non-migrant. Entrepreneurship support programs can benefit from the findings by Meister and Mauer (2019) by providing access to network and resources to fill the gaps in structural constraints that migrant entrepreneurs experience in their host country, with the long-term aim of helping participants achieve transnational entrepreneurship.

### 7.3 Implications for policy makers

This study provides some thoughts for further investigation of key issues in the field which can help academics, policymakers, and practitioners, such as: 1) how to measure the levels of human and social capital that are optimal, prior to providing financial support? 2) how to incorporate human and social capital into entrepreneurial support programs? 3) how to balance human and social capital with the financial needs (employment/entrepreneurship) of migrants, within the integration processes of new arrivals into host countries? 4) how to help migrants become aware of the options open to them upon arrival into their host countries that will allow them to enhance human and social capital and allow them to reach their potential in their host countries to integrate culturally, economically, and socially? 5) how to properly assess migrants for their extent of human and social capital to help them fill in the gaps and/or support them in their efforts to become entrepreneurs? And 6) how to introduce a bottom-up approach to take into consideration the needs and wishes of the migrants themselves to support them on their entrepreneurial journeys?

### 7.4 Implications for future research

Moving forward, this review can also help scholars to formulate new research questions that contribute to the development of the fields of migration and entrepreneurship support, which will become ever more relevant as people continue to move to Europe or change countries within Europe. It is of merit to note that three of the sources also raise the question about whether the very construction of ‘migrant entrepreneurship’ creates an otherness which perpetuates stereotypes via the comparison to local/native entrepreneurs. There is a danger of lumping immigrants together in one group despite their uniqueness and individual differences ( Högberg *et al.,* 2016; Mason, 2008), which can create an in-group/out-group scenario ( Rashid and Cepeda-García, 2021). An additional three sources (see Table 8) also touched on this by pointing out that uniform integration policies are not effective and that individual needs, including home country origins, need to be taken into consideration ( Bijedic and Piper, 2019; Hartmann and Schilling, 2018; Ram *et al.,* 2013). Moving forward, future research could investigate these issues and examine how to address the needs of migrant entrepreneurs while recognizing the uniqueness of each individual.

Our findings allowed us to propose a research agenda for practitioners and scholars to consider, along the lines of 1) facilitating human and social capital, 2) information sharing, 3) assessing human and social capital, 4) entrepreneurial support programs, 5) in-group / out-group bias in entrepreneurial support, and 6) a bottom-up approach to migrant entrepreneurship support. Table 10 provides an overview of potential research questions for these six research directions. Along the lines of point 1, facilitating human and social capital, the issue is to contribute to entrepreneurship outcomes which lead to employment and economic growth, rather than illegal entrepreneurship activities or low-margin businesses where migrant entrepreneurs compete only on price and work extremely long hours with low pay. As for point 2, information sharing, the key is helping migrants understand options available to them, that will help them learn the local language, customs, rules, regulations, educational and training opportunities, both inside and outside of entrepreneurship support. Regarding point 3, the concern is with how to assess the human and social capital of migrants to provide them with opportunities to help them build human and social capital in the host country. An example of a tool to help assess social capital is the Position Generator by Chen and Tan (2009), to gain an overview of a migrant’s network quality in both the home and host countries. Human capital can be assessed not only by diplomas and certifications, but also by testing for skillsets required for certain forms of employment and entrepreneurship. Concerning point 4, entrepreneurial support programs, there can be issues where attendees lack human and social capital, as well as motivation, to succeed at entrepreneurship. This is why it is important to assess for these factors and consider restricting access to entrepreneurial support programs unless certain conditions are met. With respect to point 5, several of the studies in our SLR raise concern that migrants are lumped into one category instead of being treated as individuals. It is worth investigating the extent that migrant entrepreneurship initiatives perpetuate stereotypes and create an in-group/out-group phenomenon, and to look at cases where migrants and natives can benefit from participating together in the same cohort of an entrepreneurship support program. As for point 6, since there is not a one-size-fits-all approach to entrepreneurial support, it would merit studies that include approaches such as co-creation ( Torfing, Sørensen, & Røiseland, 2016; Van Praag, 2021), that adopt the input of the migrants themselves into how to design an entrepreneurial support program to best suit their needs.

**Table 10.** Research Agenda.

Research Direction	Research Questions
Facilitating human and social capital	1. How to measure the levels of human and social capital that are needed prior to offering financial support to aspiring migrant entrepreneurs? 2. How to effectively establish human and social capital in migrants who lack it and aspire to become entrepreneurs? 3. How to incorporate human and social capital into entrepreneurship support programs?
Information sharing	1. How to help migrants become aware of their options after they arrive in the host country? 2. How can we operationalize the kinds of information that migrants need to settle into their host country and start a business?
Assessing human and social capital	1. How to assess human and social capital of migrants arriving at host countries? 2. How to consider each migrant’s individual needs, goals, ambitions, and potential? 3. How to consider the role of the home countries on entrepreneurial potential?
Entrepreneurial support programs	1. Which aspects of entrepreneurial support programs are specific to all entrepreneurs, and which are unique to migrants? 2. To what extent does home country matter in the content of an entrepreneurial support program? 3. Should admission to entrepreneurial support programs be restricted to those with high enough levels of human and social capital?
In/Out group bias	1. How do migrant entrepreneurship support initiatives contribute to the in group/out group bias? 2. How do migrant entrepreneurship support initiatives perpetuate stereotypes? 3. How do migrant and natives in the same cohort of entrepreneurship support programs benefit from joint participation?
Bottom-up approach	1. How to implement a bottom-up approach to migrant entrepreneurship support programs? 2. How much can the migrants themselves be involved, or co-create entrepreneurship support programs? 3. When is it beneficial for migrants to attend the same entrepreneurial support programs as natives to the host country and when should they attend dedicated programs for migrants?

**Table 11.** Characteristics of the 91 studies included in the SLR, with the codes from tables 7, 8, and 9.

Code	Authors and Years	Host Countries	Research type	Source Type	Home Country and Key Traits	Adopted theory
S1	Nijhoff, 2021	Netherlands	Qualitative	Article	Refugees	Economic Theory - Embeddedness Theory - Achievement Theory
S2	Al, De Rosa, and Perito (2021)	Italy	Qualitative	Article	India	Push-Pull Theory
S3	Barth and Zalkat (2021)	Sweden	Qualitative	Article	Refugees	No explicit theory
S4	de Lange, Berntsen, Hanoeman, and Haidar (2021)	Netherlands	Qualitative	Article	Syrian Refugees	No explicit theory
S5	Grubbström and Joosse (2021)	Sweden	Qualitative	Article	Various	No explicit theory
S6	Andersson (2021)	Sweden	Quantitative	Article	Refugees	No explicit theory
S7	Rashid and Cepeda-García (2021)	Germany	Qualitative	Article	Various	Self-categorisation theory
S8	Backman, Lopez, and Rowe (2021)	Sweden	Quantitative	Article	Refugees	No explicit theory
S9	Ozasir-Kacar and Essers (2021)	Netherlands	Qualitative	Article	Turkish females	Discursive institutionalism
S10	Solano (2021)	EU and OECD members	Quantitative	Article	Various	Mixed Embeddedness
S11	Wang and Chen (2021)	France	Mixed Methods	Article	China	Simultaneous Embeddedness
S12	Berntsen, de Lange, Kalaš, and Hanoeman (2021)	Netherlands	Qualitative	Article	Various	Embeddedness
S13	Qin (2021)	Europe	Qualitative (Secondary Data)	Article	Refugees	Cognitive Dissonance Theory
S14	Noor (2021)	United Kingdom	Qualitative	Book Chapter	Various	Mixed Embeddedness
S15	Yeasmin and Koivurova (2021)	Finland	Mixed Methods	Article	Various	Corporate Social Responsibility theory of traditional enterprises
S16	Colombelli, Grinza, Meliciani, and Rossi (2021)	Italy	Quantitative	Article	Various	Role identification theory
S17	A. Harima, Freudenberg, and Halberstadt (2020)	Germany	Qualitative	Article	Refugees	A Theory-Building Approach on its own (case study design) for emerging categories and theoretical codes
S18	Ratten and Pellegrini (2020)	Portugal	Qualitative	Article	Female	Theory Building approach with case study design
S19	Lintner and Elsen (2020)	Italy	Qualitative	Article	Refugees	No explicit theory
S20	Barth and Zalkat (2020)	Sweden	Qualitative	Article	Various	"The Liability of Newness" (the opposite of Embeddedness) - focus on 'Virtual Embeddedness' (Facebook groups and special marketing websites) as well as other strategies to overcome the 'liability of newness'
S21	Osaghae and Cooney (2020)	United Kingdom	Theory Exploration	Article	Various	Immigrant Enclave Theory
S22	Webster and Zhang (2020)	Sweden	Qualitative	Article	Female	Feminist Economics Framework
S23	Embiricos (2020)	Germany	Qualitative	Article	Refugees	Process of theory development during the course of the research
S24	Aki Harima and Freudenberg (2020)	Germany	Qualitative	Article	Refugees	Theory-building approach for constructing causalities
S25	Wade (2020)	Belgium, Netherlands, and United Kingdom	Qualitative	Article	Various	The EU entrepreneurship competence framework (EntreComp) defines entrepreneurship
S26	Szczygiel, Nunes, and Ramos (2020)	Portugal	Qualitative	Conference Paper	Various	No explicit theory
S27	Murphy, Bogue, and O'Flaherty (2020)	Ireland	Qualitative	Conference Paper	Various	No explicit theory
S28	Glinka and Hensel (2020)	Belgium, Netherlands, Poland, and the United States	Qualitative	Article	Various	Proposes a theoretical framework for studying immigrant entrepreneurship imitation decisions.
S29	Selçuk and Suwala (2020)	Germany	Qualitative	Article	Turkey	Combines theoretical approaches on entrepreneurship, family firms and migrant/ethnic economies - mixed embeddedness is included to examine the challenges of balancing socio-spatial embeddedness
S30	Bruzelius (2020)	European Union members	Qualitative	Article	European Union	Alexander (2003)'s framework for comparing and understanding local policy reactions to immigration, based on temporary/permanent
S31	De Luca and Ambrosini (2019)	Italy	Qualitative	Article	Female	Disadvantage Theory and Blocked Mobility Theory
S32	Bijedic and Piper (2019)	Germany	Quantitative	Article	Various	Embeddedness, Human Capital, and Cultural Theories
S33	Mawson and Kasem (2019)	United Kingdom	Qualitative	Article	Syrian Refugees	Theory of the entrepreneurial event model and theory of planned behavior + labor market disadvantage theory and blocked mobility hypothesis
S34	Meister and Mauer (2019)	Germany	Qualitative	Article	Refugees	Mixed Embeddedness
S35	Hagos, Izak, and Scott (2019)	United Kingdom	Qualitative	Article	Eritrea	Theory of Planned Behaviour
S36	Birdthistle (2019)	Ireland	Mixed Methods	Article	Various	COSME (Competitiveness of Small and Medium-sized Enterprises): EU policy to include access to: finance, markets, and to create a framework to encourage entrepreneurship
S37	Johnson and Shaw (2019)	Germany and Netherlands	Qualitative	Article	Syrian Refugees	No explicit theory
S38	Bolzani (2019)	Italy	Mixed Methods	Book Chapter	Various	Theory of Planned Behaviour
S39	Evansluong and Ramírez-Pasillas (2019)	Sweden	Qualitative	Article	Various	Inductive case to build a theory on the role of family social capital in host/home countries in immigrant entrepreneurs’ opportunity creation
S40	Eimermann, Mattsson, and Carson (2019)	Sweden	Qualitative	Article	Various	A conceptual framework that contrasts immigrant entrepreneurs' business and lifestyle priorities with public sector responsibilities and development interests
S41	Villares-Varela, Ram, and Jones (2018)	United Kingdom	Qualitative	Article	Various	Mixed Embeddedness
S42	Martín-Montaner, Serrano-Domingo, and Requena-Silvente (2018)	Spain	Quantitative	Article	Various	RUM framework (random utility maximization)
S43	Hartmann and Schilling (2018)	Germany	Qualitative	Book Chapter	Male Syrian Refugee	No explicit theory
S44	Villares-Varela (2018)	Spain	Qualitative	Article	Latin America/Female	Translocational positionality
S45	Williams and Krasniqi (2018)	Various western European Union countries, Norway, Switzerland, and the United States	Quantitative	Article	Kosovo	Human Capital Theory|Social Capital Theory
S46	Bolzani and Boari (2018)	Italy	Quantitative	Article	Various	Theory of Planned Behaviour
S47	Eroğlu (2018)	Western Europe	Quantitative	Article	Turkey	Disadvantage Theory and Assimilation Theory
S48	Marchand and Dijkhuizen (2018)	Netherlands	Qualitative	Book Chapter	Refugees	No explicit theory
S49	Bashir (2018)	United Kingdom	Qualitative	Book Chapter	Pakistan and Bangladesh/females	Institutional Theory and Collective Identity
S50	Basit (2017)	United Kingdom	Qualitative	Article	Pakistan/females	Social Embeddedness
S51	Ong and Freeman (2017)	Italy	Qualitative (secondary data)	Book Chapter	China	Guanxi, Confucianism and Embeddedness
S52	Munkejord (2017a)	Norway	Qualitative	Article	Female	Mixed Embeddedness
S53	Munkejord (2017b)	Norway	Qualitative	Article	Russia/females	Embeddedness
S54	Högberg, Schölin, Ram, and Jones (2016)	Sweden	Qualitative	Article	Various	Categorization and labeling as a theoretical framework
S55	Violaris, Harmandas, and Loizides (2016)	Cyprus	Qualitative	Book Chapter	Female	No explicit theory
S56	Halkias, Arifeen, and Mourad (2016a)	United Kingdom	Qualitative	Book Chapter	Pakistan/females	Blocked upward mobility
S57	Harkiolakis, Caracatsanis, Abadir, and Mourad (2016)	France	Qualitative	Book Chapter	Female	No explicit theory
S58	Halkias, Caracatsanis, Harkiolakis, Thurman, and Akrivos (2016c)	Greece	Quantitative	Book Chapter	Female	No explicit theory
S59	Abbasian and Yazdanfar (2015)	Sweden	Quantitative	Article	Various	No explicit theory
S60	Baklanov, Rezaei, Vang, and Dana (2014)	Denmark	Quantitative	Article	Various	Theory of Transnational Entrepreneurship
S61	Brzozowski, Cucculelli, and Surdej (2014)	Italy	Quantitative	Article	Various	Theory of Transnational Entrepreneurship
S62	Valenzuela-García, Molina, Lubbers, García-Macías, Pamplona, and Lerner (2014)	Spain	Mixed Methods	Article	India	Ethnic Enclave Theory and Mixed Embeddedness
S63	Vadnjal (2014)	Slovenia	Qualitative	Article	Albania	Embeddedness - push/pull theory - ethnic enclave
S64	Bouk, Vedder, and Poel (2013)	Netherlands	Qualitative	Article	Morocco and Turkey	Social Capital/Social Identity theory and Mixed Embeddedness
S65	Nathan and Lee (2013)	United Kingdom	Quantitative	Article	Various	Economic Theory
S66	Ram, Jones, Edwards, Kiselinchev, Muchenje, and Woldesenbet (2013)	United Kingdom	Qualitative	Article	Various	Mixed Embeddedness and “Super-Diversity”
S67	Kourtit, Nijkamp, and van Leeuwen (2013)	Netherlands	Quantitative	Article	Morocco	Three frameworks: 1) guest worker model 2) assimilation model 3) ethnic minority model
S68	De Noni, Ganzaroli, Orsi, and Pilotti (2013)	Italy	Quantitative	Article	Various	Mixed Embeddedness
S69	Yazdanfar and Abbasian (2013)	Sweden	Quantitative	Article	Various	No explicit theory
S70	Rezaei (2011)	Denmark	Quantitative	Article	Various	Embeddedness
S71	Riddle, Hrivnak, and Nielsen (2010)	Netherlands	Qualitative	Article	Various	The authors develop a theoretical model following a case-study approach
S72	Piperopoulos (2010)	Greece	Qualitative	Article	Various	Blocked upward mobility - opportunity structures - ethnic resources - cultural thesis
S73	Thandi and Dini (2010)	European Union members	Qualitative - secondary data only	Article	Various	Mixed Embeddedness
S74	Hammarstedt and Shukur (2009)	Sweden	Quantitative	Article	Various	Home Country Self-Employment Hypothesis
S75	Baycan-Levent and Kundak (2009)	Switzerland	Quantitative	Article	Turkey	No explicit theory
S76	Sahin, Nijkamp, and Rietdijk (2009)	Netherlands	Quantitative	Article	Morocco, Suriname and Turkey	Theory of the Need to Perform
S77	Toledano, Urbano, and Ribeiro (2009)	Spain	Qualitative	Article	Venezuela	Economic Institutional Theory
S78	Mason (2008)	Sweden	Qualitative	Book Chapter	Female	No explicit theory
S79	Wahlbeck (2007)	Finland	Qualitative	Article	Turkey	Enclave economies
S80	Lyon, Sepulveda, and Syrett (2007)	United Kingdom	Qualitative	Article	Refugees	Embeddedness
S81	Bagwell (2006)	United Kingdom	Qualitative	Article	Vietnam	Mixed Embeddedness
S82	Kontos (2003)	Germany	Qualitative	Article	Various	Grounded Theory, Network theory, Theory of Ethnic economy, Blocked Mobility
S83	Leung (2003)	Germany	Qualitative	Article	China, Taiwan, Hong Kong, Singapore, Malaysia, Vietnam	Mixed Embeddedness
S84	Lazaridis and Koumandraki (2003)	Greece	Qualitative	Article	Various	Disadvantage Theory and Embeddedness
S85	Van Delft, Gorter, and Nijkamp (2000)	Belgium, Denmark, Germany, Netherlands, Sweden, United Kingdom	Quantitative	Article	Various	No explicit theory
S86	Najib (1994)	Sweden	Qualitative	Book	Various	Disadvantage Theory, Blocked Mobility Theory and Social Embeddedness theory
S87	Yazdanfar, Abbasian, and Brouder (2015)	Sweden	Quantitative	Article	Various	Human Capital Theory, Social Capital Theory
S88	Rueda-Armengot and Peris-Ortiz (2012)	Spain	Quantitative	Article	Various	The authors provide an overview of numerous theories and integrate them: 1) Personal Attributes Theory 2) Self-efficiency (social system theory) 3) Theory of Social Learning 4) Role Model Theory 5) Block Mobility/Disadvantage Theory 6) Theory of Social Change 7) Theory of interaction 8) Cultural/Contextual/Integral
S89	Širec and Tominc (2017)	Belgium, Bosnia-Herzegovina, Croatia, Czechia, France, Germany, Hungary, Luxembourg, Netherlands, Slovakia, Slovenia, Sweden, United Kingdom	Quantitative	Article	Refugees	Theory of Planned Behaviour
S90	Kordestani, Sattari, Peighambari, and Oghazi (2017)	Sweden	Qualitative	Article	Various	Modern Stakeholder Theory & Transaction Cost Economics
S91	Andoh, Berrones-Flemmig, and Dornberger (2019)	Germany	Mixed Methods	Article	Ghana	Push-Pull Theory

## Data Availability

The data for this article consists of bibliographic references, which are included in the References section. University of South-Eastern Norway: PRISMA and PRISMA for abstracts checklists for ‘Migrant entrepreneurship in Europe: a systematic literature review’. https://doi.org/10.23642/usn.23925102. ( Polychronopoulos and Nguyen Duc, 2023). Data are available under the terms of the Creative Commons Zero “No rights reserve” data waiver (CC0 1.0 Public domain dedication).
