# Supplementary material for: Migrant entrepreneurship support in Europe: a PRISMA systematic literature review
Source: F1000Res. 2024 Jan 22;12:1300. Originally published 2023 Oct 11. [Version 2] doi: 10.12688/f1000research.139670.2 (PMC10691309; doi:10.12688/f1000research.139670.2)
Supplement: Peer review history

# Reviewer report: Reviewer response for version 2

**DOI:** 10.5256/f1000research.159868.r239586

**Published:** 2024-04-13

**Authors:** Surangi H.A.K.N.S.

**Affiliations:** 1 University of Kelaniya, Kelaniya, Western Province, Sri Lanka

Thank you for submitting your revised manuscript. I appreciate the time and effort you’ve put into addressing the comments and suggestions from the initial review.

 Upon reviewing your revisions, I am pleased to see that you have made substantial improvements to the manuscript. The changes have enhanced the clarity of your arguments and the overall quality of your work.

 Wish you all the best.

Are the rationale for, and objectives of, the Systematic Review clearly stated?

Yes

Is the statistical analysis and its interpretation appropriate?

Yes

Are sufficient details of the methods and analysis provided to allow replication by others?

Yes

Are the conclusions drawn adequately supported by the results presented in the review?

Yes

Reviewer Expertise:

NA

I confirm that I have read this submission and believe that I have an appropriate level of expertise to confirm that it is of an acceptable scientific standard.

# Reviewer report: Reviewer response for version 1

**DOI:** 10.5256/f1000research.152966.r216752

**Published:** 2023-11-23

**Authors:** Surangi H.A.K.N.S.

**Affiliations:** 1 University of Kelaniya, Kelaniya, Western Province, Sri Lanka

The topic is good. The abstract is well-written, and the article is well-structured. This is an excellent article.

 The area suggested in the topic is interesting and promising, however, the research issue is not clearly identified at the outset. Lack of explanation for the research issue and lack of support for the knowledge gap (trying to establish) are the main issues in the introduction. Therefore, the introduction section should be strengthened with elaborations of the knowledge gap focused on this study, and it should be supported by the existing literature. The introduction section should elaborate on why this review is required in this area of research. Further, the introduction section lacks the justification for the research, i.e., how the findings of this study would be beneficial to any stakeholders.

**Methodology:**

 Presenting the method of the study (the complete process as well as descriptions of each step) under the ‘research method’ section is crucial in systematic literature reviews (SLRs) in order to strengthen the validity and reliability of the data (literature) collected. The Methods section of this paper provides a clear and detailed description that is sufficient to serve this purpose.

**Contribution:**

 Thus, this paper clearly presents the outcome of the review in line with avenues for future research agendas which can be justifiable on the practical, theoretical, and methodological ground, and a detailed description that is sufficient to serve this purpose.

Are the rationale for, and objectives of, the Systematic Review clearly stated?

Yes

Is the statistical analysis and its interpretation appropriate?

Yes

Are sufficient details of the methods and analysis provided to allow replication by others?

Yes

Are the conclusions drawn adequately supported by the results presented in the review?

Yes

Reviewer Expertise:

Entrepreneurship

I confirm that I have read this submission and believe that I have an appropriate level of expertise to confirm that it is of an acceptable scientific standard, however I have significant reservations, as outlined above.

# Response

**Published:** 2024-01-17

**Authors:** Dimitris Polychronopoulos

**Affiliations:** University of South-Eastern Norway, Norway

Dear Sarangi H.A.K.N.S.

 Thank you for taking the time to review our systematic literature review. Below you will find the actions we took to address the issues you raised to us:

 Positive feedback: 

- Response: Thank you for the positive feedback on the abstract, writing composition, and structure of the article.  
- Action: None required.  

Elaborations of the knowledge gap and why this review is required in this area of research and justification for the research: 

- Response: Thank you for pointing out the need for us to more clearly identify the research issue and to provide a more robust explanation of the knowledge gap, and for highlighting the lack of justification for the research and for emphasizing the importance of explaining how the study's findings would be beneficial to stakeholders. 
- Action: We have strengthened the introduction section by providing a clear justification for the research, highlighting its potential benefits to stakeholders.  We have revised the introduction section to clearly identify the research issue and provide detailed support for the knowledge gap by linking these two concerns to the supporting literature, by adding the following paragraph to the bottom of section 1.1:  

 

 An increase in people moving to Europe coincides with the demographic trends that require immigration to fulfill labor needs and support the pensions of those who will soon be retiring and those who have already retired (Marois, Bélanger, and Lutz, 2020). Some new arrivals express an interest in becoming entrepreneurs. For example 7% of Ukrainian refugees who settled in Norway report that they aim to become entrepreneurs (Hernes, Deineko, Myhre, Liodden, and Staver, 2022) and the European Commission has incorporated refugee entrepreneurship into its 2020 Entrepreneurship Action Plan and the European Union Qualification Directive 2011/95 (article 26 and 34) states that EU members must allow access to self-employment and consider the specific needs of refugees within integration programs (Lange, Berntsen, Hanoeman, and Haidar, 2021). This SLR sees a knowledge gap surrounding the literature to support migrants of all kinds in their entrepreneurship journey, whether refugee or others. Considering that a literature review on entrepreneurship support by Ratinho, Amezuca, Honig, and Zeng (2020) shows a lack of defined evaluation of the outcomes of entrepreneurial support, this SLR aims to contribute by adding conceptual clarity to the body of literature on migrant entrepreneurship support in Europe. Therefore, with the objectives in the next section, we seek to map out the research issues on this topic. The need for this SLR is based on the context of the demographic trends of Europe, the increase in immigration to Europe, the desire of a portion of the immigrants to Europe to become entrepreneurs, and a lack of understanding surrounding the effectiveness of migrant entrepreneurship support in European contexts (Chilova, Farny, and Salmivaara, 2018; De Noni and Ganzaroli (2013); Lillevik and Sønsterudbråten, 2018), as well as a lack of trust by some migrants in government-run migrant entrepreneurship support programs (Ram, Theodorakopoulos, and Jones, 2008; Mwaura, 2019), and the call by Dheer (2018) for scholars to study policies for their impacts on migrant entrepreneurship. 

# Reviewer report: Reviewer response for version 1

**DOI:** 10.5256/f1000research.152966.r216744

**Published:** 2023-11-13

**Authors:** Robert Kloosterman

**Affiliations:** 1 University of Amsterdam, Amsterdam, The Netherlands

This is another systematic literature review on migrant entrepreneurship adding to the already existing six – four of which were published in the past three years. This could be seen as sign of the maturing of the field of research on migrant entrepreneurship and, given the multifaceted nature of the topic, there is, in principle, ample room for such meta studies. This study aims at focusing on three questions in a selected part of the literature: 1) the characteristics of migrant entrepreneurs; 2) the challenges they face; and 3) the reported policies. This study limits itself to studies regarding Europe.

 The study is well-written (a few typos) and the methodology is spelled out in detail – almost too much in detail in my view. I could have done without the many references about the discussions between “the main author” and “the second author”. I did miss at least one article that would fall within the selection criteria of the study. The study provides a useful overview of the selected articles in an accessible way. Notably the information on the geographical distribution of the studies and the growth of these over time (graph on p.13) is helpful.

 I am, however, not entirely convinced of the overall added value of this meta study. First, the study is limited to Europe. There are, obviously, differences along many dimensions in the institutional context between European countries. Still, it would have made sense to group these into say three of four groups on the basis of welfare state arrangements and migration policies as these two features have a significant impact of migrant entrepreneurs. This could then form the basis of a much more in-depth analysis of the studies they look at and can, for instance, be linked to the outlier position of Sweden.

 Second, the formulation of the three “research questions” is rather awkward. The characteristics of the migrant entrepreneurs are not so much characteristics but *issues* that the studies focus on. Many of these can indeed be traced back to Bourdieu, but also to Granovetter, Waldinger, Portes and few other pioneers. The “challenges” of the second question are, unsurprisingly, closely linked to these issues (two sides of the same coin), whereas the reported policies also, of course, are mainly targeted to these. It would, then, makes sense to regroup the trawl and position the issues as the foundation for policy making. This would also make room for much more in-depth analysis of policy making instead of just reiterating insights, like on p.35, that have already been stated in many other publications (“To this extent it is important to individualize the entrepreneurial support offered rather than lumping migrant entrepreneurs into one single group” and “how to measure the levels of human and social capital that are optimal, prior to providing financial support”- what would optimal look like: optimal in the short term, long term; in which real-life context?).

 Third, one way of really contributing to the discussion, would be to look at the changes over time. Do we see shifts in the issues and in the type of policies? Do we see geographical patterns regarding these as well. From a meta study, you would expect more than just repeating what other studies have already stated. Link the findings to a broader context. Where, in short, is the meta view in this contribution?

Are the rationale for, and objectives of, the Systematic Review clearly stated?

Yes

Is the statistical analysis and its interpretation appropriate?

Yes

Are sufficient details of the methods and analysis provided to allow replication by others?

Yes

Are the conclusions drawn adequately supported by the results presented in the review?

Partly

Reviewer Expertise:

NA

I confirm that I have read this submission and believe that I have an appropriate level of expertise to confirm that it is of an acceptable scientific standard, however I have significant reservations, as outlined above.

# Response

**Published:** 2024-01-17

**Authors:** Dimitris Polychronopoulos

**Affiliations:** University of South-Eastern Norway, Norway

 Acknowledging Positive Feedback: 

- Response: Thank you for recognizing detailed presentation of our methodology. We appreciate your positive remarks about the accessibility of the overview and the valuable insights provided, especially regarding the geographical distribution and growth of studies over time. Thank you for recognising the multifaceted nature of the topic and that there is ample room for such meta studies. With our narrow focus on support and Europe, we hope to contribute to a specific niche within this field. 
- Action: None required. 

Recognition of the multifaceted nature of migrant entrepreneurship:  

- Response: Thank you for recognizing the multifaceted nature of migrant entrepreneurship and that although the research field of migrant entrepreneurship is maturing, there is ample room for meta studies such as this one.  
- Action: To clarify how this systematic literature review is different from the other six literature reviews on migrant entrepreneurship, and to emphasize the search criteria of the study, we have added the word support to the title and in multiple parts of the text, where we felt that the stress on this specific context was missing. In addition, we added PRISMA to the title to differentiate this study from other literature reviews on migrant entrepreneurship. Given the uniqueness of the PRISMA protocol, its inclusion in the title distinguishes our study on the level of protocol and can thus help future researchers who are looking for a systematic literature review that follows the PRISMA protocol.  

Some typos throughout the article: 

- Response: Thank you for pointing out that there are some typos in the article. 

- Action: In the new version, we removed the error where the reference to Bourdieu appeared on Table 6, and we also noticed typos such as: entrepreneurship instead of entrepreneur in sections 4.2 and 5.1.1. We also removed the word ‘first’ from the end of the first paragraph in section 4.2., and corrected electronical to electronic in the last paragraph of section 3.1 We also corrected the typos in Table 9, and in sections 3.4, 4.1, and 6. 

Excessive references about the discussions between “the main author” and “the second author”.  

- Response: Thank you for bringing this issue to our attention. 
- Action: We have addressed this issue by removing references to the first and second author, with replacements such as ‘we’, ‘the authors’, and the passive voice. 

Institutional Contexts within Europe concerning Welfare State Arrangements and Migration Policies: 

- Response: Thank you for your suggestion to categorize European countries based on welfare state arrangements and migration policies to help provide a more in-depth analysis to cover the impact that such arrangements and policies have on migrant entrepreneurship, which could possibly explain the outlier of Sweden in terms of its early appearance in the field as well as its prolific representation in the literature stream.  
- Action: In consideration of the unique migration policies and welfare state arrangements of Sweden, we have looked through the sources in our SLR that focus on the Swedish context and found several unique features that are worthy of mention. Therefore, have added to the text in section 4 where we shared the results about Sweden’s early entrance and volume of studies. In addition, we provide further detail about the investigations from the other top countries in the SLR: United Kingdom, Germany, the Netherlands, Italy, and Spain. The new version now contains the following text: 

 

 With Sweden as an outlier both in terms of its early arrival into the migrant entrepreneurship support literature, as well as with its volume of studies over time, it is worth looking into possible reasons for its prolific position. First of all, Sweden has high immigration rates compared to other European countries (Evansluong and Ramírez Pasillas, 2019). Backman, Lopez, and Rowe (2020) state that Sweden had the largest share of asylum seekers between 2002 and 2013, which is one aspect that could help explain the large volume of literature covering the Swedish context. Secondly, Sweden has specific integration policies that emphasize new arrival’s first job (Webster and Zhang, 2020), along with a rigid labor market and generous support system (Backman, Lopez, and Rowe, 2020), with government organizations providing dedicated advice about entrepreneurship to immigrants (Andersson, 2021; Högberg, Schölin, Ram, and Jones, 2016; Yazdanfar, Abbasian, and Brouder, 2014).  A third factor is the change in immigration rules in 2015, where Sweden stopped recognizing credentials from the Middle East, thus resulting in higher rates of self-employment amongst impacted new arrivals (Barth and Zalkat (2021). 

 When considering Table 3 for geographical patterns, taking the top six countries, which each have at least five sources, in this SLR of 91 sources, which are dedicated to a single country: Sweden, United Kingdom, Germany, the Netherlands, Italy, and Spain, it can be useful to compare these countries to gain further insights. A common theoretical thread across all six countries is that mixed embeddedness (Kloosterman, van der Leun, and Rath, 1999) appears frequently to help provide an analytical framework for the studies. On the other hand, there are some areas of research which do not seem to have a common thread across countries. For example, exclusive coverage of refugee entrepreneurship is absent from the Spanish literature, whereas exclusive coverage of female entrepreneurship is absent from the German literature. In-depth discussion on the topic of ethnic enclaves is also absent from the German sources, although ethnic communities and ethnic markets do provide context in the article by Bijedić and Piper (2019) and co-ethnic networks in the article by Meister and Mauer (2019) as well as ethnic communities, networks, and resources by Kontos (2003). It is also noteworthy that only Italy and the United Kingdom have articles published about tech startups. The United Kingdom seems to have the highest rate of migrant entrepreneurship, with their rate being three times higher than native-born British (Osaghae and Cooney, 2020). Investigations into entrepreneurship in the agricultural sector appeared in the studies from Germany, Italy, and Sweden, while research on internationalization or transnational entrepreneurship appeared only in the articles from Italy and the Netherlands. Both Germany and Sweden have government organizations exclusively focusing on migrant entrepreneurship, and both countries have literature expressing concern of the ‘othering’ of migrants, creating an in-group/out-group and expectations of inferiority of migrant entrepreneurship compared to non-migrant entrepreneurship (Högberg, Schölin, and Ram, 2016; Mason, 2008; Rashid and Cespeda-Garcia, 2021).  

 Formulation of the Research Questions: 

- Response: Thank you for sharing your concerns about the awkward formulation of our research questions, where characteristics are simply issues that are one side of the coin and the challenges are the other, with the policies targeted toward these. We also appreciate that you pointed out that we neglected to include pioneers besides Bourdieu, who also studied these issues, such as Granovetter, Portes, and Waldinger. 

 

- Action I: We have included key pioneers in the field with the addition of the following text in Section 5.1.1: 

 

 Previous entrepreneurship literature follows the line of thought from Bourdieu (1984) by looking at the impacts of forms of capital on the establishment and maintenance of a business. Inspired by the cultural, economic, social, and symbolic capital of Bourdieu (1984), the studies covered in this SLR show a shift into three distinct forms of capital that frequently appear often in assessment of starting and running a business: financial, human, and social capital ( Baklanov et al., 2014; Eroğlu, 2018; Grubbström and Joosse, 2021). The concept of social capital is also inspired by Granovetter (1973) whose seminal work, The Strength of Weak Ties, researches how bridges across social networks relate to social mobility and social cohesion. Basit (2017) also cites Granovetter (1985) and Uzzi (1997, 1999) to share how social embeddedness is a framework by which to study economic activity, from which researchers investigate how social relations shape entrepreneurial activities and outcomes. Several sources in the SLR stress the importance of support mechanisms that build up both weak and strong ties, including Bouk, Vedder and Poel (2013), Harima et al. (2020), and Noor (2021). Waldinger (1995) also played an important role in earlier literature on social capital in migrant entrepreneurship, with his study on the construction industry in New York City, and is cited sources in the SLR by Bagwell (2008) and Barth and Zalkat (2020). This SLR uses the categories of financial, human, and social capital to classify the characteristics gathered from the studies. The literature stream considers primarily the positive impacts of the social capital from one’s ethnic group, as we did not encounter many pitfalls mentioned by Portes and Landolt (2000). However, the study by Andersson (2021), which is included in this SLR, does mention that entrepreneurship by new arrivals can depend on the extent of entrepreneurial engagement by co-ethnics already in the host-country. Andersson (2012) and another study included in the SLR, by García, Molina, and Lubbers (2014) also mention that ethnic enclaves can slow down the integration process and new arrivals’ ability to learn the host country’s culture and language.  

  

- Action II: We added to the paragraph that introduces the research questions to help establish the rest of the study as a basis for analysis of policymaking. The new text reads as follows: “This study aims at synthesizing current knowledge on migrant entrepreneurship support in Europe, by understanding the key issues that migrants in Europe face in their entrepreneurship endeavors and how these issues relate to key policy-making decisions. The study seeks to develop a conceptual framework and identify research gaps that call for future inquiry. From the above objectives, we will address three research questions (RQs) in this study, which we divide into characteristics, challenges, and support mechanisms. 

1. RQ1: What are the characteristics of migrant entrepreneurs investigated in primary studies, in the European context? 

 2. RQ2: What do we know about challenges that migrants face as entrepreneurs in European host countries? 

 3. RQ3: What do we know about reported policies as support mechanisms for migrant entrepreneurship in the European context? 

 To the best of our knowledge, this work offers a most up-to-date and comprehensive view to discern migrant entrepreneurship support in the European context and positions the issues uncovered as the foundation for policymaking.” 

 Changes over time: 

- Response: We appreciate your call to explore shifts over time as issues and types of policies evolve, and to identify geographical patterns as well, to link the findings to a broader context. 
- Action: We have enhanced the study's meta perspective by incorporating the most prominent changes over time to ensure a deeper contribution and a connection to the broader context. The following text now appears in Section 4.1: 

As for a temporal analysis of the literature, it is worth mentioning sharing our insights on how the literature has evolved over the years. First of all, the earliest source focusing on support for female migrant entrepreneurs was in 2008 by Mason. It was not until 2016 that female migrant entrepreneurship support sources appeared again. From that point, it appears regularly in the literature. See Table 11 for a full overview of the sources focusing on female migrant entrepreneurship. In addition, the first refugee entrepreneurship support study appeared as a quantitative investigation in 2017 by Širec and Tominc. There has been a steady increase since then, with 14 of the most recent 56 sources in this SLR dedicated to refugees. Table 11 shares the specific authors and publication years. Most of the migrant entrepreneurship support studies on females and/or refugees in Europe have been qualitative, with only two quantitative refugee studies, the one mentioned by Širec and Tominc and the study by Andersson (2021), and one quantitative female study by Halkias et al (2016). In addition, there has been an increase in quantitative methods over time. The first quantitative study appeared in 2000 by van Delft, Gorter, and Nijkamp, followed by three studies in 2009 and more steady appearances since 2011. 

# Reviewer report: Reviewer response for version 1

**DOI:** 10.5256/f1000research.152966.r216746

**Published:** 2023-11-13

**Authors:** Monder Ram

**Affiliations:** 1 Professor of Small Business, Centre for Research in Ethnic Minority Entrepreneurship, Aston University, Birmingham, UK

The authors aim to contribute to extant academic and policy studies by examining the characteristics and challenges of migrant entrepreneurs in Europe. The authors adopt a well-established framework to ensure that the systematic literature review is based on a comprehensive and structured synthesis of available research on migrant entrepreneurship in Europe.

 The manuscript is well-structured and comprehensive. The authors identify three research questions and have addressed them systematically. The article is clearly written and easy to follow. The authors also use relevant and contemporary sources to support their arguments.

 There are some areas where the article could be improved. Firstly, the authors could provide more information on limitations of their study. It warrants a separate section, rather than a brief mention in the conclusion. More information on the implications of the findings would be helpful too.

 Overall, the authors make a useful valuable contribution to the field of migrant entrepreneurship in Europe. They identify gaps in the field and offer helpful guidance for future research.

Are the rationale for, and objectives of, the Systematic Review clearly stated?

Yes

Is the statistical analysis and its interpretation appropriate?

Not applicable

Are sufficient details of the methods and analysis provided to allow replication by others?

Yes

Are the conclusions drawn adequately supported by the results presented in the review?

Yes

Reviewer Expertise:

Migrant entrepreneurship

I confirm that I have read this submission and believe that I have an appropriate level of expertise to confirm that it is of an acceptable scientific standard.

# Response

**Published:** 2024-01-17

**Authors:** Dimitris Polychronopoulos

**Affiliations:** University of South-Eastern Norway, Norway

 Positive Feedback: 

- Response: Thank you for recognizing the structured and comprehensive nature of our systematic literature review. We appreciate your positive comments on the clarity, systematic approach, and use of relevant sources, as well as your positive assessment of our contribution to the field of migrant entrepreneurship in Europe.  
- Action: None required. 

Limitations of the research: 

- Response: Thank you for suggesting that the discussions surrounding the limitations of the study merit a dedicated section.  
- Action: We have converted Section 5.4 into Section 6 to emphasize that the limitations merits a dedicated section. In addition, we have expanded the discussion of the limitations with the following three additions to the text: 

“Indeed, during the open review process, we received feedback that the reviewer was aware of an article that met our search criteria but still did not appear in the SLR.” 

 “An additional limitation of this study is that it does not account for the impact factor of the journals included, which is a measure that the systematic review by Aliaga-Isla and Rialp (2013) achieved. Nor does this study engage in citation analysis to focus on the most impactful articles, which is a measure taken in the systematic review by Sithas and Surangi (2021).” 

 “In addition, Table 11, which lists adopted theories of the 91 studies included in this SLR, does not list every implicit theory that the studies incorporated into their research, nor does it provide an in-depth analysis of how the theories listed are applied. Such an in-depth analysis could be worthy of future research because it would provide an overview of how scholars are applying theory and how theory application relates to research methodologies and the unit of analysis for the studies reviewed.” 

 Implications of the findings: 

- Response: Thank you for pointing out the need for a more thorough exploration of the implications of our findings. 
- Action: Considering that Section 7 of the original version included implications for policy makers and implications for future research, we have expanded Section 7 to include a section for owners of migrant entrepreneurship support programs. It reads as follows: 

 

 7.2. Implications for practitioners 

 For those running entrepreneurship support programs, it is important to include clear goals for the programs and to include metrics to evaluate their effectiveness according to the desired goals of the program. A takeaway from this SLR is that improved business performance would be a logical desired outcome for entrepreneurship program participants. So setting up ways to track improved business performance in terms of number of employees and profits are examples of metrics that can accomplish that. Other considerations are the catalysts that help generate improved business performance. Indications are that transnational entrepreneurship, defined as business taking place across national borders (Drori, Honig, and Wright, 2009) would be worth instilling into entrepreneurship support program participants long-term plans because transnational enterprises are more profitable than other forms of migrant entrepreneurship (Solano, 2016). The IntEnt non-profit incubator investigated in the Riddle, Hrivnak, Nielsen (2010) had the uniqueness of being dedicated to transnational diaspora entrepreneurship. Wang and Liu (2015) found that transnational firms have higher payroll per employee than other firms, regardless of whether the other firms are migrant or non-migrant. Entrepreneurship support programs can benefit from the findings by Meister and Mauer (2019) by providing access to network and resources to fill the gaps in structural constraints that migrant entrepreneurs experience in their host country, with the long-term aim of helping participants achieve transnational entrepreneurship.
